# Electrode Materials for High-Performance Sodium-Ion Batteries

**DOI:** 10.3390/ma12121952

**Published:** 2019-06-17

**Authors:** Santanu Mukherjee, Shakir Bin Mujib, Davi Soares, Gurpreet Singh

**Affiliations:** Department of Mechanical and Nuclear Engineering, Kansas State University, Manhattan, KS 66503, USA; sbmujib@ksu.edu (S.B.M.); soares@ksu.edu (D.S.)

**Keywords:** sodium ion batteries, anodes, cathodes, 2D materials, MXenes, alloys, prussian blue, NASICON

## Abstract

Sodium ion batteries (SIBs) are being billed as an economical and environmental alternative to lithium ion batteries (LIBs), especially for medium and large-scale stationery and grid storage. However, SIBs suffer from lower capacities, energy density and cycle life performance. Therefore, in order to be more efficient and feasible, novel high-performance electrodes for SIBs need to be developed and researched. This review aims to provide an exhaustive discussion about the state-of-the-art in novel high-performance anodes and cathodes being currently analyzed, and the variety of advantages they demonstrate in various critically important parameters, such as electronic conductivity, structural stability, cycle life, and reversibility.

## 1. Introduction

### 1.1. Current Global Energy Scenario

The progressively increasing demands for energy to meet the needs of the steadily growing population has led to huge global increases in the consumption of fossil fuels [1]. However, this large demand and ever-growing consumption of fossil fuels has led to significant undesirable consequences, primarily the large quantities of greenhouse gases (e.g., CO_2_) being liberated into the atmosphere as a result of the combustion of these fuels, leading to global warming and climate change [2,3]. Another challenge is the gradual dwindling of fossil fuel reserves, with only coal expected to last approximately another 100 years, and oil and gas for a much shorter time, which raises important questions about energy security in the long term [4,5]. As a result, a concerted effort is being taken to move away from fossil fuels and toward renewable energy providing systems (e.g., solar, wind, water (hydroelectricity), and geothermal energy) [6]. These energy systems have the advantage of being abundant (almost inexhaustible), inexpensive, and most importantly, produce no greenhouse gases [7,8]. However, most of these renewable sources, such as solar and wind, are intermittent, and, therefore, robust, reliable, and economically viable storage systems are necessary. Different energy storage types exist, such as pumped-hydro (accounting for 98% of U.S. energy storage), thermal energy storage, and compressed air [7,9]. However, electrochemical energy storage systems, by virtue of their longevity, low establishment and capital expenditure, and ease of set-up, are considered the best candidates for energy storage from renewables [9,10]. Among these rechargeable electrochemical storage systems, lithium ion battery (LIB) systems have come to play a pivotal role [11]. 

### 1.2. Current Perspective on LIBs for Energy Storage 

Ever since they were first commercialized by Sony in 1991, LIBs have transformed the way electrochemical energy is stored [12,13]. The consistent specific energies around 120 Wh kg^−1^, low reduction potentials (−3.04 V vs. SHE), and rather small ionic size of Li (0.76 Ẵ), which facilitates smooth intercalation and fast electrochemical kinetics, are some of the important aspects that make LIBs the gold standard in rechargeable metal ion electrochemical storage systems [14,15].

These advantages notwithstanding, certain aspects of LIBs need closer scrutiny. Most importantly, Li resources are rather rare in the earth’s crust (~20 ppm), and coupled with its uneven geographical availability, this scarcity makes for unfavorable LIB economics [16]. Secondly, economical constraints hinder the application LIBs in large and very large scale storage systems, such as grid storage [17]. Also, another important concern regarding LIB systems is environmental, as the usage of certain electrodes that are toxic and the flammability of certain electrolytes have been reported [18]. 

With this perspective in mind, alternative rechargeable battery systems are being studied to overcome the said challenges with lithium-based systems. It is in this scheme that sodium ion batteries (SIBs) fit in, and it is hoped that they will carve out a niche of their own in the greater metal-ion based rechargeable electrochemical energy storage ecosystem. 

### 1.3. Rationale for SIBs for Energy Storage

SIBs provide several unique advantages and are, therefore, increasingly studied as a feasible alternative to LIBs [19]. One of the most important factors is the abundant availability of Na metal in the earth’s crust (it is the 6th most abundant element), consequently making its procurement and processing relatively inexpensive [20]. Also, SIB systems have good performance in aqueous systems, unlike their LIB counterparts, and this greatly helps to bring down costs as inexpensive electrolytes and less complicated fabrication processes can be used [20,21]. Another advantage is that SIB systems, to a large extent, mimic LIB systems in their electrochemical working, which proves beneficial from a theoretical modeling standpoint, as a considerable body of knowledge already exists for LIBs [22]. All these factors lead to greater potential for SIBs to be applied in systems where large quantities of sodium are required, making them prime candidates for grid scale storage [21]. 

Despite these advantages, SIB systems still have not been able to be commercialized as several important hurdles still remain. Firstly, the large size of the Na^+^ ion (1.02 Ẵ) makes for considerably sluggish kinetics [23,24]. Also, this large ionic size results in significant constraints for smooth intercalation in the host electrode interstices, often resulting in large and undesirable volume changes, thereby reducing cycle life and lowering longevity and performance [24,25]. Scientists and researchers have been adopting different approaches to overcome these challenges, and one of the most important avenues ahead for SIB systems is from the perspective of materials engineering, i.e., developing newer and better electrodes with novel morphologies to enhance performance and longevity [26,27]. 

The main parameters to evaluate the high performance of an electrode material are broadly classified into the four categories: (a) energy density, (b) rate capability, (c) cycleability, and (d) thermodynamic stability [28]. Energy density is defined as the product of average operating potential (V) and the total amount of charge transfer (Ah) and is expressed in Wh g^−1^/Wh L^−1^. In general, for a high-performance electrode, it is desired that the electrode materials undergo fast charge and discharge while maintaining high-energy density. On the other hand, the ability of the anode material to reversibly cycle Na-ions with the least irreversible capacity (IRC) is the cycleability of the material. To achieve thermodynamic stability and prevent the possible change in anode structure, the addition of external matrix material or similar chemical modifications are desired for a high-performance electrode material.

This manuscript, therefore, devotes itself to providing a substantial discussion on the current state-of-the-art anodes (negative electrodes) and cathodes (positive electrodes) used for high performance SIB systems, as well as the different types and classes of materials used and their motivation, novel fabrication techniques, their performance, the advantages and disadvantages of each, and what future research aims to be in this regard. 

## 2. Anodes

With respect to anode materials for Na-ion batteries, it is a major challenge to develop high performance anode materials with a high reversible capacity, stable cycling performance, and high rate capability. In this review, the development of high performance of anode materials (carbons, alloy-based materials, oxides, and 2D materials) for Na-ion battery systems are discussed. The strategies to improve electrochemical performance in terms of materials fabrication, surface modification, electrolyte optimization, applying a favorable voltage window, and electrochemical performance are summarized. 

### 2.1. Carbons

Carbon based materials have been studied extensively for SIBs because of their sustainability and natural abundance. Graphite had been studied for Na ion batteries in the 1980s, but it did not allow significant Na^+^ insertion and its capacity was much lower. Therefore, soft carbons (SCs), like petroleum coke, carbon black, pitch, and PVC, were tested during the 1990s, but also showed limited capacities [29,30,31]. Later in 2011, the interest of Na-ion batteries was renewed with the study of Na intercalation in hard carbon [32]. Since then, many families of hard carbons, such as sucrose, cellulose, wood, argan shells, and peanut shells, have been studied for use as anodes, and their performance reached beyond 300 mAh g^−1^. Also, interest in soft carbons (SCs) has been growing in recent years [33,34,35,36]. However, considerable work remains in improving carbon-based anodes for Na-ion batteries.

Carbon-based materials are insertion-based materials which insert/extract a certain amount of Na^+^ during charge/discharge process. However, graphitic carbons hinder the intercalation of Na-ions due to their large radius [37]. A minimum interlayer distance of ~0.37 nm is required for Na-ion insertion, whereas the interlayer spacing of graphite is ~0.335 nm [23]. In contrast, other alkali metals, such as K, Rb, Cs, etc., are found to intercalate into graphite, even though they have larger ionic sizes than Na [38,39]. K-ion based Graphite intercalation compounds (GICs) have shown the ability to adsorb molecular hydrogen and have good conductive properties [38]. The authors have reported the successful formation of stage-I KC_8_ [38]. Alkali metals are shown to become less stable as ion size decreases from Cs to Na because of reduced ionic bonding. Although, Li is an exception, as it forms covalent bonds with C, resulting in negative formation energy. For this reason, graphite is a good intercalation material for Li and K but not for the Na [39].

Jache and Adelhelm showed that, using co- intercalation phenomena in a diglyme-based electrolyte, results in a stage-I ternary intercalation compound with an estimated stoichiometry of Na(diglyme)_2_C_20_ and the electrode showed a superior cycle life with capacities close to 100 mAh g^−1^ at a current density of 37.2 mA g^−1^ for 1000 cycles and coulombic efficiencies >99.87% [40]. Wen et al. utilized another strategy by expanding the interlayer distance of graphite to 0.43 nm through a process of oxidation and partial reduction on graphite and reported a high reversible capacity of 284 mAh g^−1^ at 20 mA g^−1^ and retained 73.92% of its capacity at 100 mA g^−1^ after 2000 cycles [37]. 

Mesoporous soft carbon (MSC) was prepared by Cao et al. form mesophase pitch using nano-CaCO_3_, which consists of a disordered region with a large interlayer distance [35]. As an anode in Na-ion batteries, this MSC delivered a reversible capacity of 331 mAh g^−1^ at a current density of 30 mA g^−1^ and retained a capacity of 103 mAh g^−1^ at 500 mA g^−1^ after 3000 cycles. Recently, Yao et al. fabricated microporous soft carbon nanosheets (SC–NS) through microwave assisted exfoliation [41]. According to the authors, micropores and defects at the edges of as-prepared SC–NS contributed to enhanced kinetics and extra Na storage sites and exhibited a high reversible capacity of 232.2 mAh g^−1^ at 1000 mA g^−1^ and 128.7 mAh g^−1^ at 800 mA g^−1^ after 3500 cycles.

Hard carbons have also been utilized as anodes for Na-ion batteries due to their disordered structure and large interlayer distance [42]. Further, Hard carbon can deliver high capacity and low voltage plateau near 0.1 V (vs. Na/Na^+^) [43,44]. Luo et al. derived carbon nanofibers from cellulose nanofibers and investigated them as materials for an anode [45]. A high reversible capacity of 255 mAh g^−1^ was obtained at a current density of 50 mA g^−1^ and an excellent cycling ability of 176 mAh g^−1^ was reported at 200 mA g^−1^ for over 600 cycles. Cao et al. prepared hollow carbon nanowires (HCNWs) through the pyrolization of a hollow polyaniline nanowire precursor [23]. The HCNWs used as an anode for Na-ion batteries delivered a high reversible capacity of 251 mAh g^−1^ at 50 mA g^−1^ and showed a capacity retention of 82.2% over 400 cycles between 1.2 and 0.01 V (vs. Na^+^/Na). Li et al. also reported porous carbon nanofibers (P-CNFs) that delivered an reversible capacity of ~140 mAh g^−1^ at a current density of 500 mA g^−1^ after 1000 cycles [46]. P-CNFs were prepared by pyrolysis of PAN-F127/DMF nanofibers via an electrospinning process. Lotfabad et al. utilized pyrolyzed banana peel pseudographite (BPPG) as the anode for Na-ion batteries and reported a gravimetric capacity of 355 mAh g^−1^ at 50 mA g^−1^ and a stable charge capacity of 221 mAh g^−1^ at 500 mA g^−1^ after 600 cycles [47]. The authors attributed this performance of BPPG to the dilated intergraphene spacings for Na intercalation and near-surface nanopores. 

**Figure 1:**
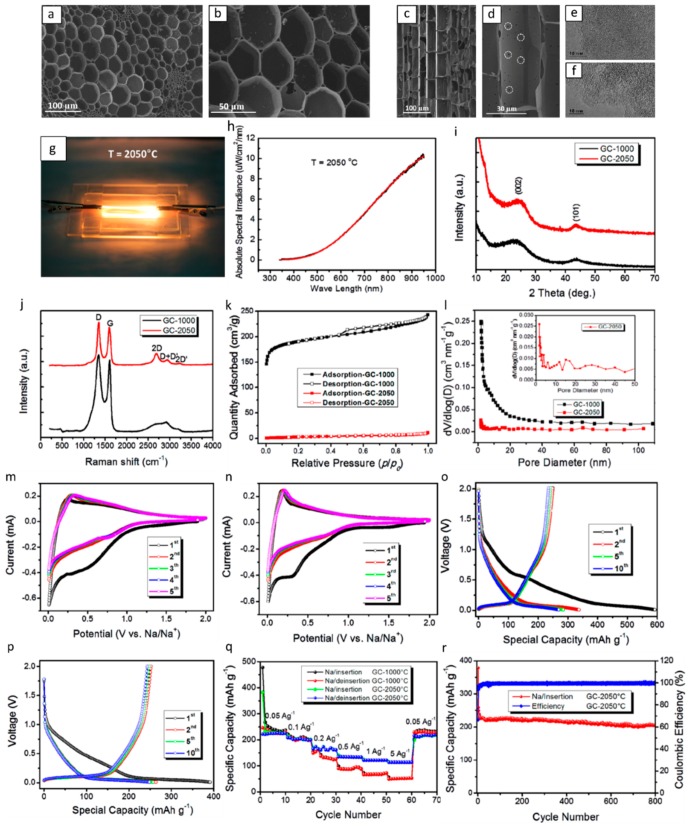
Structural characterization and electrochemical performance analysis of Hard Carbon (carbonized switchgrass, GC-1000 and GC-2050) as an anode in Sodium ion batteries (SIBs): (**a**) Low magnification SEM image from a cross section of GC-1000. (**b**) Corresponding higher magnification SEM image of GC-1000. (**c**) Low magnification SEM image from longitudinal section of GC-2050 where vertical lines are showing the array structure. (**d**) Higher magnification SEM image of GC-2050 where dashed circles highlight the micro/nanoscale holes for ion transport. (**e**,**f**) HRTEM (High-resolution transmission electron microscopy) images of GC-1000 and GC-2050. (**g**) Digital photograph of switchgrass derived carbon under Joule heating. (**h**) Light emission spectra of the carbonized switchgrass, here the temperature was fitted according to blackbody radiation equation. (**i**) XRD patterns of the GC-1000 (black) and Gc-2050 (red). Samples show identical twin broad peaks around 2ϴ = 23.6° and 43.6°, corresponding to the diffraction of graphitic layered structure. (**j**) Raman spectra of GC-1000 (black) and GC-2050 (red) confirmed the progressively ordered structure in the samples. (**k**,**l**) N_2_ adsorption/desorption isotherms and pore size distribution of GC-1000 and GC-2050, respectively. (**m**,**n**) Cyclic voltammogram (CV) curves for the first five cycles of GC-1000 and GC-2050, respectively at a scanning rate of 0.5 mV s^−1^. (**o**,**p**) Voltage profiles of GC-1000 and GC-2050 respectively at a current density of 50 mA g^−1^ in the potential range: (0.01–2 V). (**q**,**r**) Specific capacity, coulombic efficiency, and rate capability of GC-1000 and GC-2050 samples. Reprinted with permission from [48]. Copyright 2017 American Chemical Society.

For room temperature Na-ion batteries, Zhang et al. synthesized hard carbon, under an ultrahigh temperature (2500 °C), from switchgrass [48]. The authors reported that because of its three-dimensional porous hierarchical structure and large interlayer spacing of 0.376 nm, carbonized grass showed a specific capacity of 210 mAh g^−1^ as the anode of Na-ion batteries at a current density of 0.1 A g^−1^, and when cycled at 50 mA g^−1^, a stable capacity of 200 mAh g^−1^ was retained over 800 cycles with ~100% coulombic efficiency after the first few cycles. Figure 1 represents a summary of the results of this group as representative of the performance of the carbon-based anode material for high performance Na-ion batteries. Figure 1a–f shows the microporous feature of the carbonized switchgrass and highlights the array structure and micro/nanoscale holes for ion transport. Graphitic layer structures can be seen from XRD and Raman in Figure 1i–j. Figure 1k–r shows the representative cyclic voltammogram (CV), voltage profiles, and galvanostatic charge-discharge (GCD) of the carbon anodes for the 1st, 2nd, 5th, and 10th cycles, at a current density of 50 mA g^−1^ in a potential range of 0.01−2 V versus Na/Na^+^. The initial coulombic efficiencies for the GC-1000 and GC-2050 anode are calculated to be 42% and 64%, respectively. Compared to GC-1000, the capacity of GC-2050 in both the sloping and plateau regions reduced slightly, which is, according to the authors, due to a decrease of active sites of electrochemical adsorption/desorption to sodium ions. 

Large area phosphorous-doped carbon nanosheets (P-CNSs) were obtained from carbon dots (CDs) and analyzed by Hou et al. [49]. A high reversible capacity of 328 mAh g^−1^ was obtained at 0.1 A g^−1^ with a stable capacity of 149 mAh g^−1^ at a current density of 5 A g^−1^ after 5000 cycles for P-CNSs [49]. In the recent past, Hu et al. synthesized a nitrogen rich (17.72%), hierarchically porous, and highly disordered carbonaceous material (N-HC), with an enlarged interlayer distance of 0.44 nm by spray drying and pyrolysis under NH_3_ [50]. This porous and nitrogen doped N-HC was used as the anode in Na-ion batteries and showed a reversible discharge capacity of 255.9 mAh g^−1^ at 500 mA g^−1^ in the 3000th cycles [50]. A low-cost typha-derived hard carbon with acid activation was synthesized and analyzed in Na-ion batteries by Shen and coworkers [51]. They reported a high reversible capacity of 204.8 mAh g^−1^ at a current density of 100 mA g^−1^ after 4000 cycles [51]. Zhao et al. synthesized a low temperature (1300 °C) hard carbon with graphite crystals, using carbonized eggshell membranes, and sucrose-derived microsphere as precursors [52]. The hard carbon/graphite structure showed a discharge capacity of 310 mAh g^−1^ at 20 mA g^−1^ and 260 mAh g^−1^ after 1000 cycles at 100 mA g^−1^ [52].

Overall, Carbon based anodes are leading candidates for Na-ion batteries, but they need further study. Graphitic carbons limit the Na intercalation by forming a compound that is not energetically favorable, and solvation energy plays a role in intercalation, which needs to be further studied. Broad interlayer spacings of carbon can lead to a higher capacity. On the other hand, for hard carbons, it is believed that Na ion intercalations correspond to a low potential plateau. Additional study on the Na storage mechanism, especially the contribution of plateau regions, is needed to understand this intercalation. 

Table 1 lists the performance of several notable carbon-based anodes that have been used as anodes for Na-ion battery applications.

### 2.2. Alloys

Because of their high energy density and low redox potential, alloys have garnered considerable interest as anode materials for Na-ion batteries. Alloy-based materials can alloy with Na to form various Na-metal-alloy phases that contribute to higher capacities [19]. In 2011, Chevrier and Ceder reported that Si, Ge, Sn, Pb, and Sb could alloy with sodium and show theoretical capacities of 954, 369, 847, 485, and 660 mAh g^−1^, respectively [62]. However, Si was found to be nearly inactive with Na [63]. Further, the large volume expansion of the alloy anodes during the charge/discharge process can lead to poor cycle life, capacity fade, and degradation of the electrode coating [62]. A good binder, better nanostructures, and active material diluted with inactive materials are being incorporated to alloys to achieve cycling stability in Na-ion batteries [62,64]. During the last decade, researchers have started fabricating alloy based compounds for Sn, Sb, Ge, P, and some other elements from group IVA (Pb, Bi) with electrochemically inactive/active components, which help to alleviate volume variation during the charge/discharge processes and provide good cycleability [65,66,67,68].

#### 2.2.1. Sn-Based

Sn is an attractive anode for Na-ion batteries, as it can provide a theoretical capacity of ~850 mAh g^−1^ by forming Na_15_Sn_4_ [64,69]. In situ sodiation/desodiation TEM analysis of tin nanoparticles (80–400 nm) shows different phase transitions from amorphous NaSn_2_ to Na-rich amorphous phases, such as aNa_9_Sn_4_ and a-Na_3_Sn, respectively. Crystalline Na_15_Sn_4_ is finally obtained after full sodiation [70]. However, the Sn particles expand by 420% after sodiation, thus reducing the cycling performance of the anode [42]. 

Hu et al. deposited thin film of Sn on a hierarchical wood fiber substrate and showed that the soft nature of the wood fibers releases the mechanical stresses associated with the electrochemical process. Moreover, their mesoporous structure works as an electrolyte reservoir [71]. They reported an initial discharge capacity of 339 mAh g^−1^ at a current density of 84 mA g^−1^ and 145 mAh g^−1^ after 400 cycles [71]. Liu et al. prepared ultra-small Sn nanoparticles (~8 nm), homogenously embedding them in the spherical carbon network by the spray analysis method, and the as-prepared 8-Sn@C delivered an initial reversible capacity of 493.6 mAh g^−1^ at a current density of 200 mA g^−1^ and a stable capacity of 415 mAh g^−1^ after 500 cycles at 1000 mA g^−1^ [72]. According to the authors, the well-dispersed ultra-small Sn nanoparticles and the conductive carbon network contribute to the high performance of 8-Sn@C as anode in Na-ion batteries [72]. 

In addition, to alleviate the volume change, pipe-wire TiO_2_-Sn@carbon nanofibers (TiO_2_-Sn@CNFs), synthesized by electrospinning and atomic layer deposition, were analyzed as an anode in Na-ion batteries by Mao et al. [73]. A high reversible capacity of 413 mAh g^−1^ at 100 mA g^−1^ after 400 cycles was reported, which, according to the authors, was due to the formation of the amorphous Na_x_Sn phase during charge/discharge, thus allowing Sn particles well-proportioned volume expansion in Na-ion batteries [73]. Sha et al. studied Sn nanoparticles on a nitrogen-doped carbon nanofiber composite (Sn@NCNFs), synthesized by electrospinning [74]. The results of this study indicate that, because of the uniform distribution of Sn nanoparticles on the surface of the nitrogen-doped carbon nanofibers, the material delivered a high specific capacity of 600 mAh g^−1^ at a current density of 84.7 mA g^−1^ and maintained a capacity of 390 mAh g^−1^ at a high density of 847 mA g^−1^ over 1000 cycles [74]. Similarly, Pan et al. distributed Sn nanodots (3.2 nm) in a sheet-like nitrogen-doped carbon framework (Sn@SNC) and reported a high mass loading up to 20 mg cm^−2^ with a reversible areal capacity of 1.0 mAh cm^−2^ at 3 mA cm^−2^ after 700 cycles [75]. 

**Figure 2:**
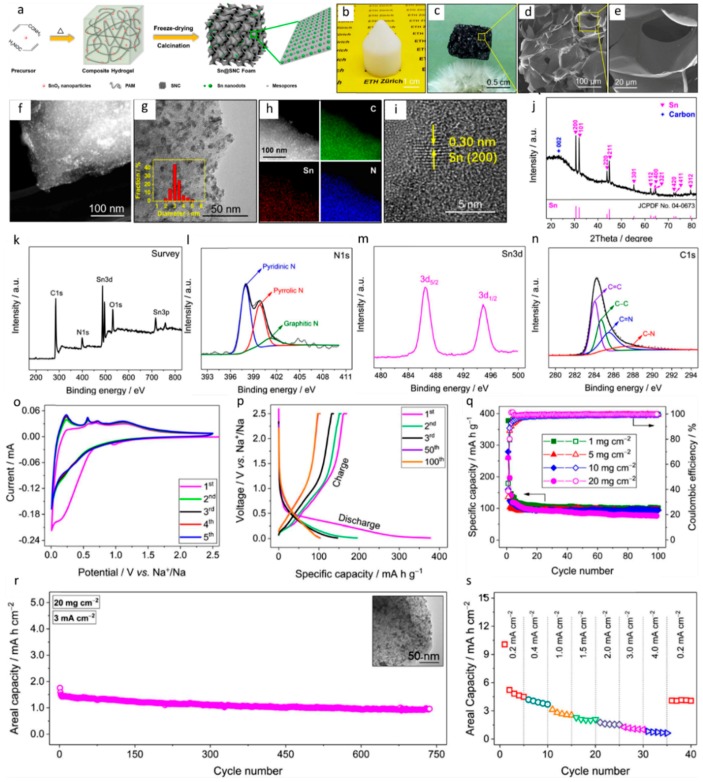
Structural characterization and electrochemical performance analysis of Sn-based alloy (Sn@SNC) as an anode in SIBs: (**a**) Synthetic procedure of Sn@SNC foam from SnO_2_/polyacrylamide composite hydrogel. (**b**,**c**) Photographs of SnO_2_/PAM composite hydrogel and hierarchically porous Sn/SNC foams. (**d**,**e**) SEM image of Sn@SNC structures show a 3D interconnected microporous structure and confirm the sheet-like carbon structure. (**f**,**g**) STEM & TEM images confirm the uniform distribution of Sn nanodots in the SNC. Inset shows the diameter of Sn nanodots are ~3.2 nm. (**h**) STEM with element mappings also confirm the homogenous distribution of the Sn nanodots throughout the SNC. (**i**) HRTEM image of Sn@SNC shows the lattice spacing of 0.30 nm, distinctive to the (200) plane of metallic Sn. (**j**) XRD patterns of the Sn@SNC shows the co-existence of metallic Sn and amorphous SNC. (**k**–**n**) XPS survey spectrum demonstrates the presence of C, N, and Sn, confirming the disordered structure and successful doping of N in SNC. (**o**) CV curves for the initial five cycles of Sn@SNC at a scanning rate of 0.1 mV s^−1^. (**p**) Voltage profiles of Sn@SNC at a current density of 100 mA g^−1^ delivering an initial discharge capacity of 377.6 mAh g^−1^ with 44.7% coulombic efficiency. (**q**) The Sn@SNC foams deliver a specific capacity of 101.8 mAh g^−1^ at a mass loading of 1 mg cm^−2^ after 100 cycles at a current density of 100 mA g^−1^. At a higher mass loading of 20 mg cm^−2^, the anode shows a capacity decrease of 25% with a specific capacity of 76.3 mAh g^−1^. (**r**) The Sn@SNC anode shows a good cycling ability, delivering a high reversible areal capacity of 1 mAh cm^−2^ at a current rate of 3 mA cm^−2^ after 700 cycles. (**s**) Rate capability of Sn@SNC foams (mass loading = 20 mg cm^−2^) at various current densities. A reversible areal capacity of 0.6 mAh cm^−2^ is achieved at a high current of 4 mA g^−1^. Reprinted with permission from [75]. Copyright 2018 Elsevier.

Figure 2 demonstrates the important findings of Pan et al.’s work in greater detail [75]. Figure 2a–c shows the synthesis of hierarchically porous Sn@SNCs, which are beneficial for achieving high Na^+^ storage. The interconnected 3D microporous structure and uniform distribution of Sn nanodots are confirmed by SEM (Scanning Electronic Microscopy) and TEM (transmission electron microscopy) in Figure 2d–i. Figure 2j–n investigates the crystallinity and co-existence of metallic Sn and SNC using XRD (X-ray diffraction), Raman, and XPS. A broad peak in the range of 0.4–0.1 V is recognized in all cathodic scans in Figure 2o, corresponding to the insertion of Na^+^ into the Sn nanodots. From the 10th cycle on, the coulombic efficiency remains unaltered (Figure 2p), demonstrating the high structural stability and sufficient conductivity of the Sn@SNC foams. The high mass loading Na^+^ storage of the hierarchically porous Sn@SNC foams is displayed in Figure 2q–s, indicating efficient diffusion of Na^+^ through thick porous electrodes.

In general, reducing the particle size of Sn to the nanoscale effectively mitigates the pulverization of the electrode materials. Sn/carbon composites or carbon coated Sn show improved electrochemical performance compared to bare Sn. The carbon matrix acts as a buffer for volume change and enhances the electrical conductivity. As an effective approach to alleviate the volume changes of Sn-based electrodes, electrically conductive CNT (carbon nanotube)-coated soft cellulose fibers are utilized to release the mechanical stress caused by the volume change of Sn during sodiation and desodiation, resulting in improved cycle performance. In addition, the porous nature of the substrates functions as a reservoir that helps in the ion transportation of electrolytes. Crystalline Sn/carbon composites formed by the carbonization of tin oxide nanoparticles and Sn nanoparticles dispersed in carbon nanofibers in an argon atmosphere have also been effective in reducing volume change and showing high capacity. 

However, Sn/carbon composites and Sn nanoparticles embedded in carbon show poorer kinetics and rate performance in Na-ion batteries than Li-ion batteries. Therefore, optimized electrolytes need to be developed along with Sn-based materials to decrease their charge transfer resistance, which results from the formation of different solid electrolyte interphases (SEIs).

#### 2.2.2. Sb-Based

In addition to Sn, Antimony (Sb) is also a promising alloy anode for Na-ion batteries. Sb performs better as an anode in Na-ion batteries than in Li-ion batteries, which is believed to be due to the formation of an amorphous intermediate phase of Na_3_Sb [19,76]. Allan et al. used operando pair distribution function (PDF) analysis and ex situ ^23^Na magic-angle spinning solid-state nuclear magnetic resonance (MAS ssNMR) to explain the alloying mechanism of high capacity SB anodes for Na-ion batteries [77]. The authors identified two intermediate species: a-Na_(3-x)_Sb (x = 0.4–0.5), with a significant number of Na vacancies and a-Na_3_Sb, with a highly amorphous structure. According to the authors, sodium mobility within c-Na_3_Sb leads to the excellent rate capability of Sb anodes [77].

To overcome the volume changes during the charge/discharge process, Zhu et al. synthesized freestanding electrospun Sb–C nanofiber mats (SbNP@C) without any binder, carbon black, or current collector [78]. This study found that SbNP@C can deliver an initial capacity of 422 mAh g^−1^ and could be sustained 350 mAh g^−1^ after 300 cycles at a current density of 100 mA g^−1^ [78]. Similarly, Wu et al. synthesized Sb–C nanofibers through a single-nozzle electrospinning technique and calcination, where the Sb particles were distributed homogenously in the carbon nanofibers [79]. The authors reported that Sb–C nanofiber electrode as anode material of Na-ion batteries can deliver a high reversible capacity of 631 mAh g^−1^ at a current density of 40 mA g^−1^, good rate capability of 337 mAh g^−1^ at 3 A g^−1^, as well as cycling stability (446 mAh g^−1^ at 200 mA g^−1^ after 400 cycles, showing a capacity retention of 90%) [79]. 

Zhao et al. dispersed ultrafine Sb nanoparticles in the Nitrogen-rich 3D conductive carbon network structure solving the problems of pulverization, loss of electrical contact and low utilization rate [80]. This study showed that, the full cell Na_3_V_2_(PO_4_)_3_@C//C@Sb had high output potential of 2.75 V and a discharge capacity of 264.3 mAh g^−1^ at 1 A g^−1^ after 500 cycles [80]. Cui et al. utilized Sb nanorods encapsulated into highly conductive N and S codoped carbon frameworks (Sb@(N, S–C)) as an anode for Na-ion batteries and demonstrated that the Sb@(N, S–C) anode can display a high discharge capacity of 812.7 mAh g^−1^ at 100 mA g^−1^ and maintain a reversible capacity of 390.8 mAh g^−1^ after 1000 cycles at a high current of 1 A g^−1^ [81]. The authors ascribed these impressive properties to the unique cross-linked carbon networks that provide highly conductive frameworks for fast ion transport and prevents the agglomeration of Sb nanorods during the electrochemical process [81].

Overall these studies highlight the beneficial aspects of Sb nanostructures well-dispersed in carbon frameworks, improving the performance of an Sb anode in Na-ion batteries by reducing the volume expansion and providing good electrical conductivity. As has been shown for Sn, Sb-based compounds with carbon can suppress the deformation of alloy materials and enhance electrochemical performance. Further, it is important to find an optimized electrolyte for an electrode material with a large volume change to improve electrochemical performance.

**Figure 3:**
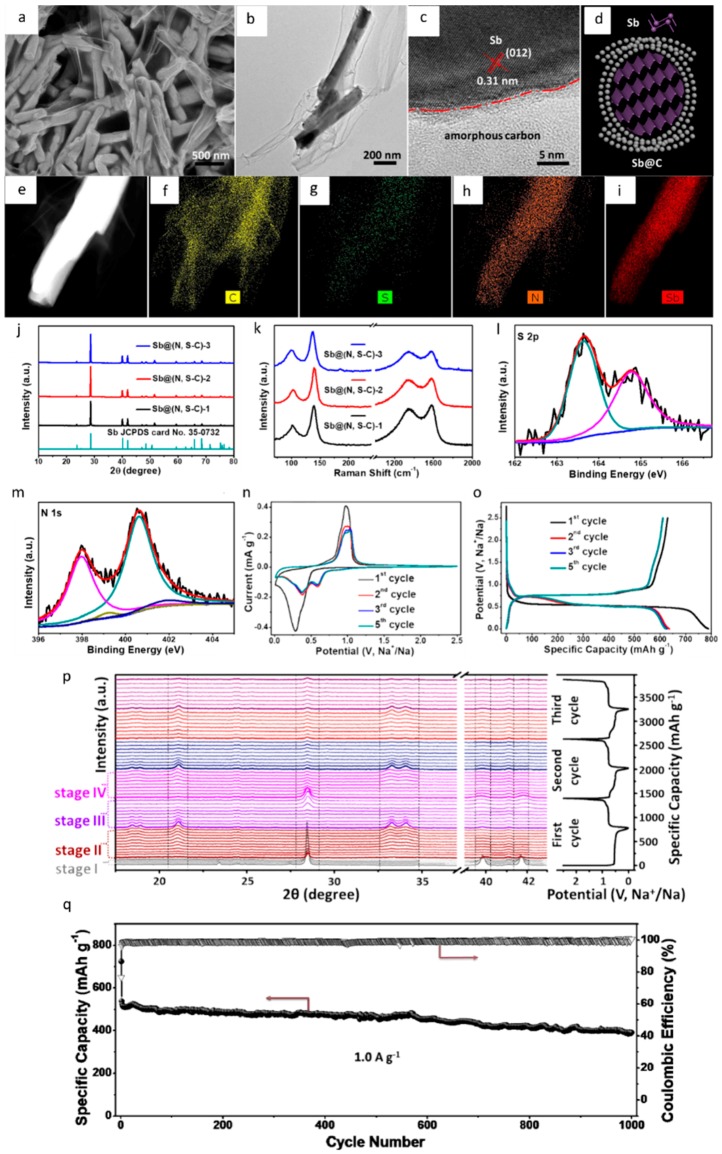
Structural characterization and electrochemical performance analysis of Sb-based alloy (Sb@(N, S−C)) as an anode in SIBs: (**a**) SEM image of Sb@(N, S−C)-2. (**b**) TEM image of Sb@(N, S−C)-2 shows Sb nanorod diameter (120–160 nm). (**c**) HRTEM image of Sb@(N, S−C)-2 reveals the amorphous carbon and clear lattice corresponding to the plane (012) of Sb. (**d**) Schematic representation of Sb@(N, S−C)-2. (**e**–**i**) Elemental mapping of Sb@(N, S−C)-2 confirm the uniform distribution of C, S, N, and Sb in Sb@(N, S−C)-2 hybrid. (**j**) XRD patterns of the Sb@(N, S−C)-1 (black), Sb@(N, S−C)-2 (red), and Sb@(N, S−C)-2 (blue) reveal the hexagonal structured Sb. (**k**) Raman spectra display the typical Sb signals (~101 and ~138 cm^−1^) and Carbon (D-band ~1343 cm^−1^ and G-band ~1581 cm^−1^). (**l**,**m**) XPS spectra of the Sb@(N, S−C)-2 Show the characteristic S 2p3/2 at ~161.7 eV and S 2p1/2 at ~162.9, which are assigned to -C-S−C- covalent bond. In addition, the existence of pyridinic N at ~398.0, pyrrolic N at ~400.7, and graphitic N at ~401.9 eV is also confirmed in the XPS peaks of Sb@(N, S−C)-2. (**n**) CV curves for the first five cycles of Sb@(N, S−C)-2 at a scanning rate of 0.1 mV s^−1^. (**o**) Voltage profiles of Sb@(N, S−C)-2 at a current density of 100 mA g^−1^ in the potential range: (0.01–2.50 V). The capacity loss in the first cycle is attributed to the SEI film formation. (**p**) In situ XRD measurements of Sb@(N, S−C)-2 for initial three cycles indicates the sodiation of Sb follows the mechanism of Sb → Na_x_Sb → Na_3_Sb → Sb. (**q**,**r**) Specific capacity and coulombic efficiency of Sb@(N, S−C)-2 sample at 1 A g^−1^. Reprinted with permission from [81]. Copyright 2019 American Chemical Society.

A summary of Cui et al.’s results is presented in Figure 3, which is representative of the performance of Sb-based anodes in high performance Na-ion batteries [81]. Figure 3a–d shows Sem and TEM images of the Sb@(N, S–C). The “silk-like” carbon networks in the Sb@(N, S−C)-2 hybrid are distributed around the Sb nanorods, which can be regarded as a highly conductive bridge for each Sb@C nanorod. the elemental mappings of Sb@(N, S−C)-2 in Figure 3e–i confirm the uniform distribution of N, S, C, and Sb, in the Sb@(N, S−C)-2 hybrid. The uniform distribution of N, S, C, and Sb are further confirmed by XRD, Raman, and XPS in Figure 3j–m. Figure 3n shows the initial five cycles of the cyclic voltammogram (CV) curves of Sb@(N, S−C)-2 at a scan rate of 0.1 mV s^−1^ between 0.01 and 2.50 V. During the first cathodic scan, a sharp peak at ~0.27 V is clearly observed, which can be ascribed to the alloy of Sb with Na forming Na_x_Sb. Figure 3o displays the galvanostatic charge/discharge curves of the Sb@(N, S−C)-2 at a current density of 100 mA g^−1^, in a voltage range of 0.01−2.50 V. In Figure 3p, the Sb@(N, S−C)-2 electrodes at different states of charge/discharge are analyzed using in situ XRD. As shown in the figure, the intensities of the three main Sb peaks become weaker in subsequent cycles, demonstrating how amorphous Sb is accumulated during the sodiation–desodiation cycles. Figure 3q shows the cycleability of the sample at a current density of 1 A g^−1^. After 1000 cycles, the sample retains a reversible capacity of 390.8 mAh g^−1^.

#### 2.2.3. Ge-Based

Recently, Ge is being studied as a promising anode material for Na-ion batteries, as Ge has shown high lithium storage capacity and high lithium diffusivity [82,83]. However, Stojić et al. showed that Na diffusion in crystalline Ge is several orders of magnitude slower than lithium diffusion in germanium [84,85]. Abel et al. showed that using nanocolumnar germanium thin films, synthesized by evaporative deposition, can cause faster diffusion in sodium [84]. They were able to achieve a high initial sodium storage capacity of 430 mAh g^−1^, and 88% of this capacity was retained after 100 cycles at a current density of 74 mA g^−1^. In addition, the nanocolumnar germanium exhibited a high rate capability of 164 mAh g^−1^ at 10 A g^−1^. Comparing the dense germanium film with nanocolumnar germanium. From the ab initio molecular dynamics simulations, the authors suggested that nanoscale dimensions are essentially stable, and a high rate capability of germanium can be observed in Na-ion batteries [84]. 

To overcome the large volume change during charge/discharge, Wang et al. synthesized germanium@graphene@TiO_2_ core-shell nanofibers (Ge@G@TiO_2_ NFs) as an anode for Na-ion batteries through electrospinning and atomic layer deposition [86]. This Ge@G@TiO_2_ NFs material, as an anode, showed an initial capacity of 368 mAh g^−1^ at a current density of 100 mA g^−1^ and retained 182 mAh g^−1^ after 250 cycles. According to the authors, graphene could naturally accommodate the volume change to release the stress in germanium, offer chemical stability, and provide more active site for sodium ion insertion. On the other hand, SEI accumulated on TiO_2_ on the outer surface instead of the Ge nanoparticles, thus increasing the stability of the anode [86].

#### 2.2.4. P Based

Phosphorous (P) has gained attention as a non-metallic anode material in Na-ion batteries because it exhibits a theoretical capacity of ~2600 mAh g^−1^ by forming Na_3_P compounds with a redox potential of 0.4 V vs. Na^+^/Na [87,88]. Kim et al. showed that the electrochemical performance of amorphous red phosphorous was dependent on volume expansion and electrical conductivity [89]. P has a low electrical conductivity of 10^−14^ S cm^−1^. The authors reported that obtaining red phosphorous/carbon composite powders by a simple ball milling process can provide a high specific capacity of 1890 mAh g^−1^, at 73% of the theoretical capacity [89]. Nearly at the same time, Qian et al. reported an amorphous phosphorous/carbon (a-P/C) nanocomposite, synthesized by high-energy ball milling at an optimized ratio of 7:3 and showed a reversible capacity of 1764 mAh g^−1^ with a first cycle coulombic efficiency of 87%, at a current density of 250 mA g^−1^ [88]. The authors showed that adding 10% fluoroethylene carbonate (FEC) in the electrolyte to enhance the stability of the SEI film on the anode can deliver ~1000 mAh g^−1^ after 140 cycles. 

Hollow nanoporous red phosphorous (HNPRP) with a little amount of iodine doping was synthesized by Liu et al. and utilized as an anode for Na-ion batteries [90]. The HNPRP electrode delivered a high capacity of 1658.2 mAh g^−1^ at a current density of 260 mA g^−1^ and an ultralong cycling ability generating 857.3 mAh g^−1^ at 2600 mA g^−1^ after 1000 cycles. The authors attributed this result to the hollow nanoporous structure that provides fast electronic transport and Na-ion accessibility [90]. Yao et al. also synthesized hollow porous carbon nanospheres with vaporization–condensation process to achieve low volume expansion and high sodium storage [91]. They reported a long cycle life of 1000 cycles with 548 mAh g^−1^ at 1000 mAh g^−1^ and a high reversible capacity of 1915 mAh g^−1^ at a current rate of 100 mA g^−1^ [91]. 

Very recently, Jin et al. proposed a ternary composite of black phosphorous (BP), graphite, and polyaniline, containing 65 wt.% of black phosphorous [92]. This BP based ternary composite provided an optimized ion pathway (electrolyte → polyaniline (PANI) → black phosphorous-graphite (BP-G) → BP), which reduced the charge transfer resistance and allowed a higher sodiation/desodiation. This ternary composite showed a reversible gravimetric composite of 1530 mAh g^−1^ at a current density of 250 mA g^−1^ and a capacity retention of 520 mAh g^−1^ at a high current density of 4 A g^−1^ after 1000 cycles [92]. 

**Figure 4:**
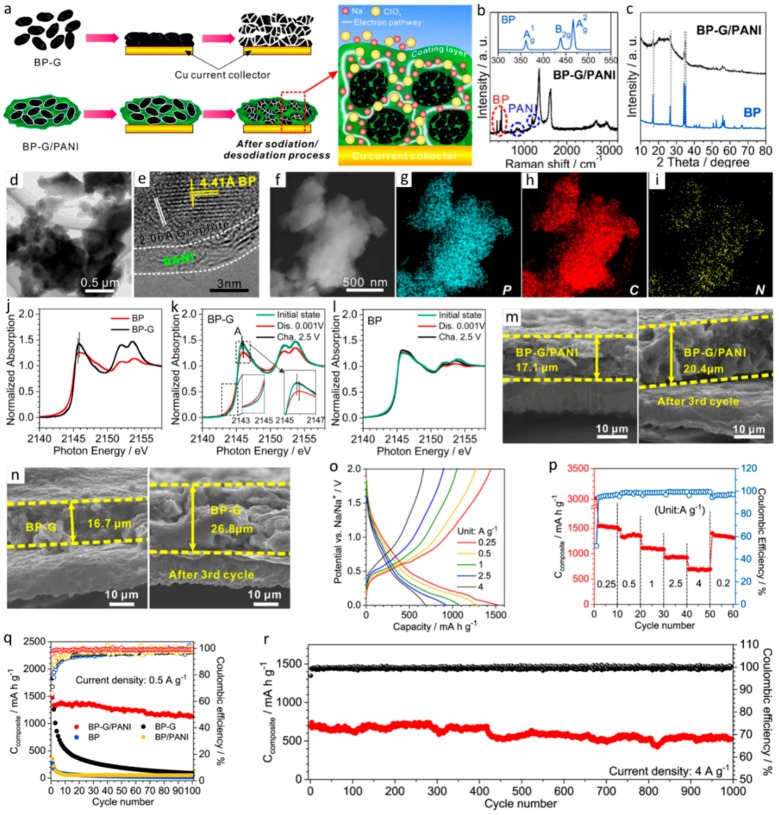
Structural characterization and electrochemical performance analysis of P-based anode (BP-G/PANI) in SIBs: (**a**) Schematic representation of sodiation process of black phosphorous-graphite (BP-G) and black phosphorous-graphite with polyaniline (BP-G/PANI). (**b**) Raman spectra shows the feature peaks of BP, graphite, and PANI. (**c**) XRD patterns show the characteristic diffraction peaks for BP at 2ϴ values of 16.8, 26.5, 34.1, and 34.9°, a broad peak for PANI at ~22°. Raman spectra and XRD patterns confirm that BP particles remain intact in the BP-G/PANI composite. (**d**) TEM image and (**e**) HRTEM image of BP-G/PANI characterize the micro-structure. TEM and HRTEM image confirm that the BP particle are well coated with PANI. (**f**–**i**) TEM mappings of BP-G/PANI confirm the uniform distribution of P, C, and N in BP-G/PANI. (**j**) X-Ray absorption spectra of P state in BP and BP-G shows higher absorption edge in BP-G corresponding to higher charge transfer. (**k**,**l**) Ex situ XAS of P K-edge of BP-G and BP indicate the formation NA_x_P by sodiation in BP-G. (**m**,**n**) Cross-sectional SEM images of BP-G/PANI electrode and BP-G electrode show that PANI coatings attenuate the volume changes in electrode, providing a long cycling ability for BP-G/PANI. (**o**) The voltage profiles of BP-G/PANI at different current densities in the potential range: (0.01–2.00 V). The capacity loss in the first cycle is attributed to the SEI film formation. (**p**) Rate capability test of BP-G/PANI anode shows that it can deliver 690 mAh g^−1^ (45% capacity retention) at high current rate of 4 A g^−1^. (**q**) The cycling stability and coulombic efficiency of BP, BP-G, BP-G/PANI at 0.5 A g^−1^ show that the reversible capacity of BP-G/PANI is mostly contributed by BP. (**r**) The cycling ability and coulombic efficiency of BP-G/PANI anode at 4 A g^−1^ show that it can deliver a reversible capacity 520 mAh g^−1^ after 1000 cycles. Reprinted with permission from [92]. Copyright 2019 American Chemical Society.

Figure 4 includes the important findings of Jin et al.’s work in details [92]. Figure 4a shows different steps in the fabrication of a ternary black phosphorous-graphite-PANI composite, where PANI offers a Na-ion pathway and the graphite reduces the ion charge transfer resistance. Raman and XRD confirm that BP remains intact in the composite in Figure 4b,c. In Figure 4d–i, the microstructure of the composite is characterized, and even the distribution of C, P, and N are shown by the HRTEM and TEM mappings. The function of graphite and PANI in the BP-based composite have been analyzed using ex-situ XAS (X-ray absorption spectroscopy) spectra and cross-sectional SEM images in Figure 4j–n. Figure 4o shows the voltage profiles of BP-G/PANI at different current densities in the range of 0.25–4 A g^−1^ within the voltage window of 0.01−2 V (vs. Na/Na^+^). The reversible capacity of BP-G/PANI is 2350 mAh g^−1^ in terms of BP mass, which corresponds to 90% BP utilization and delivers a capacity of 690 mAh g^−1^ at a higher current density of 4 A g^−1^ (Figure 4p). Similar measurements carried out for BP and BP-G electrodes as shown in Figure 4q, which shows that the reversible capacity of BP-G/PANI is almost contributed by the BP. Figure 4r proves the ultrastable cyclic capability of BP-G/PANI at a high rate of 4 A g^−1^ for 1000 cycles.

In general, therefore, it seems that high energy mechanical milling and vaporization-condensation strategies are effective processes. Phosphorous−Carbon nanostructures are well preserved and provide fast Na-ion accessibility, which leads to higher cycling performance. However, the higher the loading of P, the higher the capacity. On the other hand, the high interlayer size of black phosphorous provides high intercalation of Na ions. Further, the conductive properties of black phosphorous (~300 S m^−1^) promote electron transport within the composite. 

#### 2.2.5. Other Alloys

To date, outside of Sn, Sb, and Ge, metal alloys have not been commonly utilized as anode materials for Na-ion batteries [93,94,95]. Among them, Bi-based alloys have shown promising properties. Wang et al. showed that bulk Bi coupled with glyme-based electrolytes can achieve 400 mAh g^−1^ at a current density of 50 mA g^−1^, and the capacity retention was 94.4% after 2000 cycles with 389 mAh g^−1^ at 400 mA g^−1^ [96]. This study showed that the bulk Bi electrode can gradually become porous during initial cycling, thus ensuring facile Na^+^ transport and structural stability, which was in contrast with the Na^+^ transport and structural stability in carbonate-based electrolytes [96]. Recently, encapsulated Bi spheres with a conductive porous N-doped carbon shell (Bi@N-C) anode were synthesized by Yang et al. [97]. This Bi@N-C anode in Na-ion batteries exhibited an initial charge capacity of 327 mAh g^−1^ at 1 A g^−1^ and a high cycling capability of 235 mAh g^−1^ after 2000 cycles at 10 A g^−1^ [97].

Intermetallic composite anodes have also been investigated and show promising performance as anodes for Na-ion batteries. For instance, Ji et al. reported using porous carbon nanofiber supported SnSb nanocomposites as anodes for Na-ion batteries [98]. They showed that presence of fluoroethylene carbonate (FEC) improved the kinetics of Na ions and delivered a high reversible capacity of 350 mAh g^−1^ at a current density of 100 mA g^−1^ and a capacity retention of 99.4% for more than 200 cycles [98].

Liu et al. fabricated a yolk-shell structure for a high capacity and electrochemically stable Sn_4_P_3_ electrode surrounded by a thin carbon shell [99]. According to the authors, the void space between the C shell and Sn_4_P_3_ nanoparticles allowed volume expansion without deforming the structure. Due to this unique structure, the Sn_4_P_3_@C nanoparticles delivered a high reversible capacity of 790 mAh g^−1^ at a current density of 100 mA g^−1^ and were stable after 400 cycles, delivering 360 mAh g at 1500 mA g^−1^ [99].

Li et al. presented a carbon coated Mo_3_Sb_7_ composite (Mo_3_Sb_7_@C) as an anode material for Na-ion batteries [100]. This study showed that Mo_3_Sb_7_@C had an initial capacity of 400 mAh g^−1^ at 98.8 mA g^−1^ and maintained 338 mAh g^−1^ at 247 mA^−1^ after 800 cycles with a capacity retention of 91.8% [100].

Nanostructured iron distannide (FeSn_2_)-graphite composite was fabricated by Edison et al., and the influence of graphite concentration was studied for Na storage [101]. This composite FeSn_2_-Gr composite delivered a sodiation capacity of over 400 mAh g^−1^ at a current density of 100 mA g^−1^ and showed a high rate capability of ~200 mAh g^−1^ at high current of 1 A g^−1^ [101].

**Figure 5:**
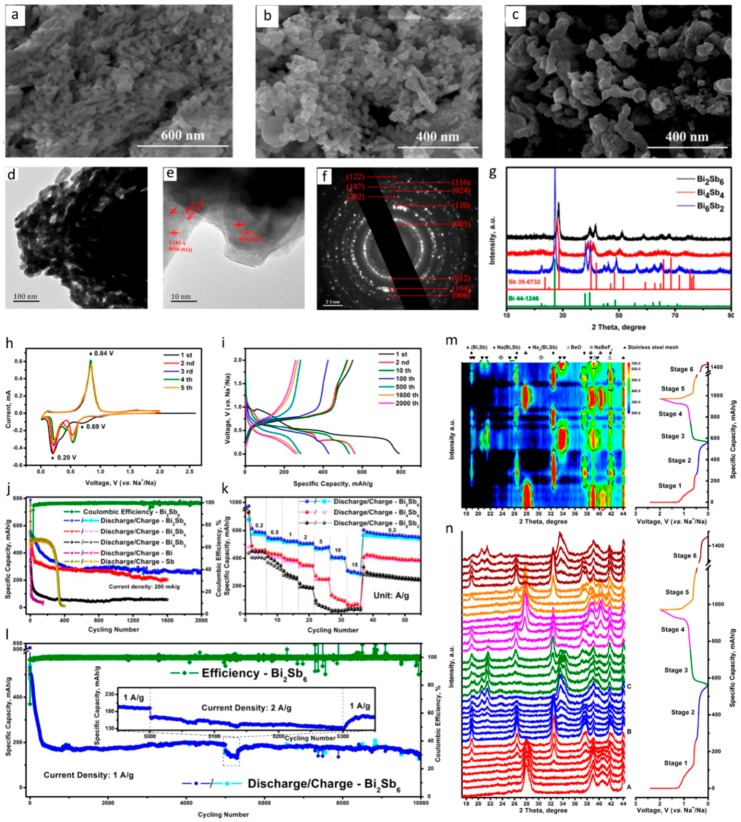
Structural characterization and electrochemical performance analysis of intermetallic alloy (np-Bi-Sb) as an anode in SIBs: (**a**–**c**) SEM images of the np-Bi_2_Sb_6_, np-Bi_4_Sb_4_, and np-Bi_6_Sb_2_ samples, respectively showing typical three-dimensional bi-continuous ligament-channel with various size of ligaments. (**d**) TEM image of the np-Bi_4_Sb_4_ features the nanoporous structure of np-Bi-Sb alloys. (**e**) The high resolution TEM (HRTEM) image shows that the crystal size (~25 nm) of the np-Bi-Sb alloys are approximately close to the ligament’s sizes. (**f**) The selected-area electron diffraction (SAED) pattern of the np-Bi-Sb alloy further confirms the nanocrystalline structure of the selected region. (**g**) XRD patterns of the np-Bi-Sb alloys are indexed to the single phase of BiSb alloy. (**h**) Cyclic voltammograms (CV) of the np-Bi_2_Sb_6_ electrode at a scan rate of 0.1 mV s^−1^ over a potential window of 0.01−2.0 V (vs. Na^+^/Na). (**i)** GCD curves of the np-Bi_2_Sb_6_ electrode in different cycles at a current density of 200 mA/g. The overlap of the charge/discharge curves of the 1600th and 2000th cycles implies the excellent stability during the long cycling. (**j**,**k**) Excellent cycling performance and rate capability of the np-Bi-Sb alloys, respectively. (**l**) Cycling performance and coulombic efficiency of the np-Bi_2_Sb_6_ electrode at 1 A g^−1^ exhibit amazing cycling capability. A zoom-in image (inset) illustrates the cycling performance at 2 A g^−1^ when the discharge capacity can remain about 150 mAh g^−1^ from 5000 to 5300 cycles. (**m**–**n**) Contour plot and line plot of the operando XRD of the np-Bi-Sb alloys during the charge/discharge processes reveals the sodiation/desodiation procedures of the alloy as the anode of the Na-ion batteries. Reprinted with permission from [102]. Copyright 2018 American Chemical Society.

In the recent past, Gao et al. fabricated a nanoporous bismuth–antimony alloy (Bi_2_Sb_6_) and tested it as an anode. This Bi_2_Sb_6_ alloy demonstrated an excellent cycling performance of 10,000 cycles at 1 A g^−1^ with a capacity retention of only 0.0072% (~150 mAh g^−1^) [102]. The authors attributed the high performance of the Bi_2_Sb_6_ to the porous structure of the alloy and the proper Bi/Sb atomic ratio. The authors also presented a sodiation process ((Bi, Sb) → Na(Bi, Sb) → Na_3_(Bi, Sb)) during the charge/discharge of the Bi_2_Sb_6_ alloy, using operando X-ray diffraction and density functional theory calculations [102]. The main results of this work are shown in Figure 5 as a representative example of the bimetallic anode’s high performance in Na-ion batteries. The scanning electron microscopy (SEM) images (Figure 5a–c) show that all three np-Bi-Sb samples exhibit typical three-dimensional bicontinuous ligament-channel structures. Transmission electron microscopy (TEM) is used to further confirm the nanoporous feature of the np-Bi-Sb (Figure 5d–f), which are in good agreement with the SEM results. In Figure 5g, the XRD patterns of the three samples are indexed to the single phase of BiSb alloy, while the minor shifts of the peaks are attributed to the compositional difference between Bi and Sb. Figure 5h shows the Cyclic Voltammetry. In the first cathodic scan, one board peak at 0.69 V (vs. Na/Na^+^) and a strong broad peak at 0.20 V (vs. Na/Na^+^) can be seen. These values are attributed to the formation of a solid electrolyte interphase (SEI) film and the two-step alloying processes of (Bi, Sb) → Na(Bi, Sb) → Na_3_(Bi, Sb), respectively. Figure 5i shows the galvanostatic discharge−charge curves of the np-Bi_2_Sb_6_ electrode in different cycles at a current density of 200 mA g^−1^ over a potential window of 0.01−2.0 V (vs. Na/Na^+^). Overlapping of the voltage profiles of the 1600th and 2000th cycles proves excellent cycle stability. Figure 5j demonstrates the comparison of cycling performances of the np-Bi_2_Sb_6_ with the np-Bi_4_Sb_4_, np-Bi_6_Sb_2_, np-Bi, and np-Sb electrodes. The rate capability of the np-Bi-Sb electrodes at different current densities are shown in Figure 5k. After 10,000 cycles, the np-Bi_2_Sb_6_ electrode still retains a moderate discharge capacity of ~150 mAh g^−1^ (Figure 5l). The phase evolution of the np-Bi_4_Sb_4_ electrode is investigated by the operando XRD technique during the initial discharge−charge processes at 25 mA g^−1^ by contour and line plot (Figure 5m,n). At the beginning of the discharge, the peaks at 28.0°, 38.9°, and 40.9° can be ascribed to the (Bi, Sb), then the appearance of peaks at 18.9°, 26.4°, 32.5°, 37.8°, and 42.1° are indexed as the Na(Bi, Sb) formation, and the peaks at 18.9°, 19.3°, 20.6°, 21.5°, 25.3°, 26.4°, 33.6°, 34.1°, 38.6°, and 39.8° are assigned to the Na_3_(Bi, Sb).

Table 2 lists the performance of several notable alloy-based anodes, which have been used as anodes for Na-ion battery applications

### 2.3. Metal Oxides

**Figure 6:**
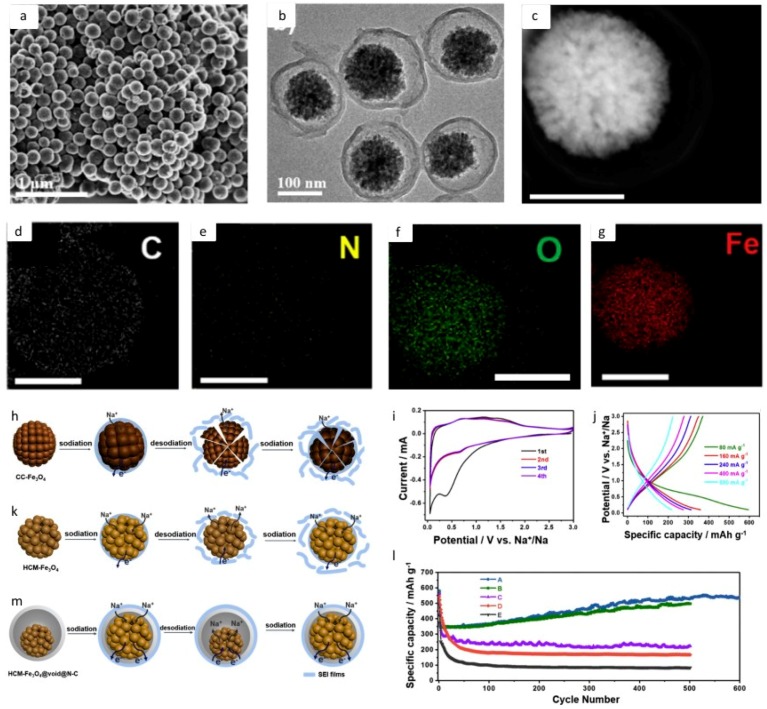
Structural characterization and electrochemical performance analysis of metal oxide yolk-shell structured HCM-Fe_3_O_4_@void@N–C nanospheres as an anode in SIBs: (**a**) field emission scanning electron microscope (FESEM) of the material, (**b**) transmission electron microscopy (TEM) of the material, (**c**) scanning transmission electron microscopy (STEM) of the material, (**d**–**g**) energy dispersive X-ray (EDX) elemental mappings of a HCM-Fe_3_O_4_@void@N–C nanosphere, (**h**) representation of the sodiation and desodiation processes of CC-Fe_3_O_4_ and their characteristic volume increases, (**i**) cyclic voltammetry curves from 1st to 4th cycles for voltage range from 0.01–3.0 V. It is worth noting that the first cycle shape differs from the other cycles because of side reactions and Solid Electrolyte Interphase (SEI) formation, (**j**) charge–discharge curves for different current densities of HCM-Fe_3_O_4_@void@N–C nanospheres, (**k**) representation of the sodiation and desodiation processes of HCM-Fe_3_O_4_, (**l**) specific capacity representation versus cycle number for current density of 160 mAg^−1^ of the following materials: (A) HCM-Fe_3_O_4_@void@N–C, (B) HCM-Fe_3_O_4_@void@C, (C) HCM-Fe_3_O_4_@C, (D) HCM-Fe_3_O_4_, and (E) CC-Fe_3_O_4_ nanospheres, and (**m**) representation of the sodiation and desodiation processes of HCM-Fe_3_O_4_@void@N–C nanospheres, showing the SEI film, which accommodates the volume expansion characteristic of Fe_3_O_4_ species. The bars of (**c**–**g**) are 100 nm. Reprinted with permission from [51]. Copyright 2019 Elsevier B.V. All rights reserved.

Because of their high theoretical capacities, metal oxides are considered promising candidates for anodes in SIB batteries. The first known report of layered transition metal oxides for SIB was published in the 1980s [115]. Within this family, Fe_2_O_3_ provides a theoretical capacity of ≈1007 mAh g^−1^—. Furthermore, Fe_2_O_3_ is an environmentally friendly and low-cost species due to its abundance [116,117,118,119]. Likewise, another example of a transition metal oxides species is Fe_3_O_4_, which presented a specific capacity of 522 mA h g^−1^ at 160 mA g^−1^ after 800 cycles [51]. In the work published by Zhao et al., in order to overcome the low conductivity of Fe_3_O_4_ and volume changes during sodiation and desodiation, the authors employed several techniques, such as a successive sol-gel process, the solvothermal method, and calcination, for instance, to obtain the material called HCM-Fe_3_O_4_@void@N–C [51]. Then, by providing a void space within the material, the authors found a way to overcome the volume changes above mentioned. Apart from this, the nitrogen-doped carbon contributed to higher electronic conductivity. Further details of this work are depicted in Figure 6. Here Figure 6a–c is the electron microscope images demonstrating the mesoporous composite nanospheres. Figure 6d–g are the EDX elemental maps demonstrating the constituent elements present uniformly. Figure 6h,k,m is the schematic representation of the sodiation of the different spheres indicating the superiority of the Fe_3_O_4_@void@NC spheres. Figure 6i,j,l are the GCD and capacity retention performance of the spheres as anodes in an SIB. Another attractive metal oxide for SIB anodes is the CuO, due to its high theoretical capacity (674 mA h g^−1^) [120], low cost, and availability. In the work developed by Wang et al., CuO quantum dots of ≈2 nm were prepared and embedded in carbon nanofibers by the electrospinning deposition technique. The results show that the material called 2-CuO@C presented specific capacity of 401 mA h g^−1^ at a current density of 500 mA g^−1^ after 500 cycles, with an initial specific capacity of 528 mAh g^−1^ at 100 mA g^−1^ [121]. Accordingly, as it can be seen, metal oxides are usually employed as SIB anodes with carbon species, such as *γ*-Fe_2_O_3_@C, 3D composite that presented a capacity of 358 mA h g^−1^ after 1400 cycles at 2000 mA g^−1^, with Coulombic efficiency near 100% [122]. In addition, MnCoNiO*_x_*@double-carbnon nanofibers presented a specific capacitance of 230 mA h g^−1^ at 100 mAg^−1^, with capacity retention of 96% for over 500 cycles. This work, developed by Wu et al., reveals another interesting result—a capacity retention of 89% after 6500 cycles (at 1000 mA g^−1^) with specific capacity of 107 mAh g^−1^ [123].

In comparison, when it comes to non-transition metal oxides, Sb_2_O_4_@rGO, in the work developed by Ramakrishnan et al., 500 cycles showed a reversible capacity of 626 mAh g^−1^ at 600 mA g^−1^ [124]. Additionally, for Sb_2_O_4_@rGO, the reactions that happen upon sodium accommodation are as follows:

Sb_2_O_4_ + 8 Na^+^ + 8 e^−^ → 2 Sb + 4 Na_2_O (1)

2 Sb + 6 Na^+^ + 6 e^−^ → 2 Na_3_Sb. (2)

Another relevant aspect is the contribution of the electrolyte for high performance sodium ion batteries. In the work published by Li et al., rhombic TiO_2_ nanocrystals were synthesized and electrochemically characterized for two different electrolytes, i.e., NaCF_3_SO_3_ in ethylene carbonate and diethyl carbonate (EC/DEC), and NaCF_3_SO_3_ in diglyme. The results show that after 500 cycles at 100 mA g^−1^, the reversible capacity for the electrolytes with EC/DEC and diglyme were, respectively, 87 mA h g^−1^ and 165 mA h g^−1^ [125,126]. A tabular listing of some other prominent results for the anodes discussed above is provided in Table 3 below.

Overall, metal oxides present high capacity values, when compared with hard carbon’s theoretical capacity, low cost, nontoxicity, chemical stability, and also environmental friendly nature [127]. Nevertheless, the literature reports that these high capacity values are usually achieved at low current densities [128]. This happens because of the low conductivity of the metal oxide species and structural changes, which require several steps and energy for such reorganization to take place [127,128]. Furthermore, another concern when it comes to metal oxide anodes are the volume changes that occur during sodium insertion and extraction, which may lead to an agglomeration of metal oxide particles and even cracks or the pulverization of the active material causing loss of contact and an impedance increase in the cell [129,130]. Those facts combined may affect the cell performance, causing capacity and rate capability to decrease [131,132]. Regarding the future perspectives of metal oxide anodes, aiming to provide capacities closer to the theoretical capacity values for metal oxides, strategies using carbon materials and nanostructuring is expected to increase the mobility of Na^+^ ions in these anodes, as well as the ability to accommodate volume expansion. Those aspects are crucial to the commercial feasibility of SIB employing metal-oxides anodes.

### 2.4. 2-D Materials

#### 2.4.1. Graphene

In 2013, for the first time, reduced graphene oxide (rGO) as an anode was reported in SIB anodes [146]. In this work, published by Wang et al., rGO was prepared by first oxidizing graphite by a modified Hummers’ method, followed by a heat-treatment. The presented capacity results for reversible Na storage in the rGO synthesized at 1.0 C (200 mA g^−1^) for the 2nd, 100th, and 250th cycles were 177 mAh g^−1^, 110.3 mAh g^−1^, and 94.3 mAh g^−1^, respectively. Moreover, for over 1000 cycles, the discharge capacity, at 0.2 C (40 mA g^−1^), was 141 mAh g^−1^, presenting, therefore, steady cycle retention [146]. These results show the improvement of sodium insertion because of rGO, thereby providing higher capacity values, even for higher current densities, when compared to other carbon allotropes. This improvement was achieved due to the existence of defects that contribute towards an increase of the graphene interlayer distance [146,147].

Over the years, graphene has gained the attention of researchers due to its unique characteristics, such as large surface-area, high electronic and thermal conductivity, high elasticity, mechanical strength, and chemical stability [148,149,150,151]. Thus, for the aspects mentioned herein, graphene is usually employed as standalone anodes and conducting agent for electrodes of SIBs due to its ability to provide short diffusion length for ions, leading to their faster diffusion, and also because of graphene’s large surface, which provides more channels for ion insertion [86,152,153,154]. 

Zhang et al. synthesized binder-free porous graphene film (PGF) electrodes for SIB, with pore sizes ranging from 38 to 450 nm [86]. For the electrochemical results, the work shows that the first reversible capacity for the PGF-1 film was 193 mA h g^−1^ at 50 mA g^−1^ in the first cycle, while at 1000 mA g^−1^ (for the 500th and 1000th cycles) the specific capacities were 156 mAh g^−1^ and 111 mAh g^−1^, respectively. 

**Figure 7:**
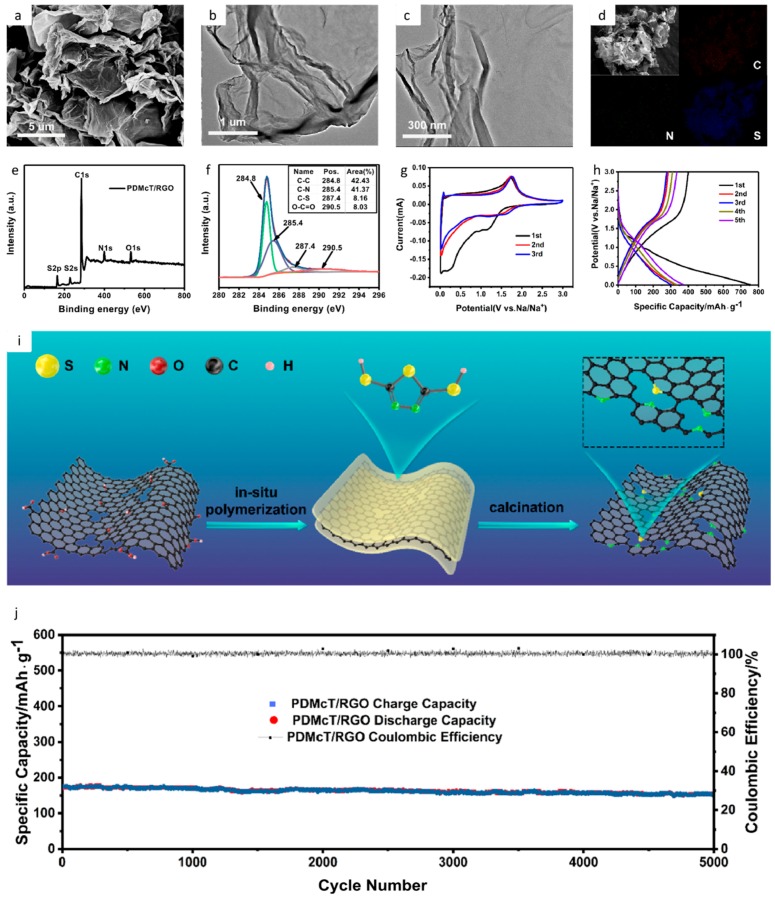
Characterization and electrochemical performance analysis of poly(2,5-dimercapto-1,3,4-thiadiazole)(PDMcT)/reduced graphene oxide (RGO) (PDMcT/RGO) as an anode in SIBs: (**a**) scanning electron microscopy (SEM) of the material, (**b**,**c**) transmission electron microscopy (TEM) of the material, (**d**) energy dispersive X-ray (EDX) elemental mapping of the material indicating the homogeneous distribution of atoms N, and S co-doping the graphene sheets, (**e**) X-ray photoelectron spectroscopy (XPS) survey of the material corroborating the results obtained at EDX, (**f**) high resolution XPS of C 1s showing the C-C, C-N, and C-S characteristic binding energies, (**g**) cyclic voltammetry curves from 1st to 3rd cycles for voltage range from 0.01–3.0 V at scan rate of 0.1 mV s^−1^. In the 1st cycle, at approximately 0.2 V, the reduction peak may be assigned to the solid-electrolyte interphase (SEI) formation; additionally, at approximately 0 and 0.09 V, redox peaks may be assigned, respectively, to Na^+^ insertion/extraction from graphene layers, (**h**) charge and discharge curves at current density of 50 mA g^−1^, (**i**) representation of the synthesis of the material PDMcT/RGO, and (**j**) specific capacity representation versus cycle number for current density of 5000 mA g^−1^ showing the low drop in capacity even after 5000 cycles; thus, remaining at 153.3 mAh g^−1^. Reprinted with permission from [155]. Copyright 2018, American Chemical Society.

Furthermore, a strategy to develop a high performance anode for SIBs is to employ doped-graphene. In recently published work [155], co-doped graphene oxide nanosheets with heteroatoms were employed. By adding a polymer containing nitrogen and sulfur, the monomer was adsorbed on the surface of graphene oxide, following a polymerization reaction. The electrochemical results for the SIB anode unveil a capacity retention of 82% after 800 cycles and, for a current density of 500 mAg^−1^, a specific capacity of 237.2 mAh g^−1^ [155]. The results of this work have been provided in Figure 7 as a representative. Here Figure 7a is the SEM image and 1b,c are the TEM images of the polymer/rGO nanosheet composite. Figure 7d–f demonstrates the elemental and chemical characterization as seen in the EDS elemental map and the XPS graph. Figure 7g–j represent the electrochemical performance of the polymer/rGO composite as an anode in an SIB system.

It is also important to highlight that, in the literature, graphene is commonly employed with other materials aiming to enhance their desirable properties. This process can be illustrated by the synthesis of SbPO_4_ and rGO, performed by Pan et al., through the solvothermal reaction of SbCl_3_ and NH_4_H_2_PO_4_ with graphene oxide, followed by a reducing atmosphere annealing with Ar and H_2_ [156]. In this work, the layered monoclinic SbPO_4_ structure was deposited on RGO aiming to enhance the electrochemical properties of the material. The results show a capacity of 100 mAh g^−1^ at 1000 mA g^−1^ and its stability up to 1000 cycles. Moreover, the energy density at 1.2 kW kg^−1^ was 99.8 Wh kg^−1^.

In summary, graphene is usually employed as a support material in SIB anodes, due to its fast electron transport and mechanical properties [157,158,159]. Nevertheless, pristine graphene presents a low value of reversible capacity for Na^+^ storage, which is the reason why graphene is usually employed with another species [158]. Furthermore, as stated by Ling et al., puregraphite is energetically unstable for intercalating Na^+^, and also for low concentrations, which is the reason why pristine graphene is not employed uniquely as active material anode for SIBs [160,161,162]. One alternative is to introduce topological defects aiming to increase the capacity and rate capability of graphene-based SIB anodes, or to use graphene as support for other 2D materials, such as Transition metal dichalcogenides (TMDs) and phosphorene (also reported herein) for example. Given the importance graphene has achieved nowadays, this material is expected to continue to have a strong presence within energy storage systems in the future, as a support material for high performance devices. 

#### 2.4.2. Phosphorene

Since 2014, Phosphorene, a single layer of black phosphorus, has recently emerged (since 2014) as a beyond graphene technologically relevant material owing to its superior mechanical and electronic properties, ease of fabrication through common low-cost exfoliation techniques. Applications of phosphorene as high capacity anode material for SIB has also been investigated. [163,164,165,166].

Therefore, although phosphorene has similarities to graphene, such as being a 2D material obtained through mechanical exfoliation of black phosphorous, graphene can be obtained through the exfoliation of graphite and possesses its layered structure in elemental form, unlike TMDs. [164,167,168,169,170]. Some of these properties include high carrier mobility (≈200–1000 cm^2^V^−1^s^−1^) and a direct band gap for one or several multi-layers (0.3 eV–2 eV) [169,170]. It is also important to highlight that phosphorene is a candidate for SIB anodes, as its interlayer spacing is 5.4 Å, which is greater than graphite (3.7 Å) [171,172]. As a result, the specific capacity of phosphorene for SIB is theoretically 2596 mAh g^−1^, almost seven times hard carbon’s specific capacity [173,174]. Sun et al. in [175] developed a phosphorene-graphene material that presented a specific capacity of 2440 mAh g^−1^ after 100 cycles at 50 mA g^−1^ (0.02 C), with a capacity retention of 83%. In this work, graphene contributed to a faster charge transfer between its layers, and improved mechanical properties, allowing the anisotropic expansion of the phosphorene layers during cycling.

To summarize, phosphorene is a material of interest because of its high capacity values and its applicability to several fields, such as optoelectronics, solar cells, and biomedicine. However, the ways of obtaining this 2D material are still under investigation, as mechanical exfoliation and liquid-phase exfoliation are typically employed. Regarding those techniques, mechanical exfoliation presents a low yield, while liquid-phase exfoliation (ultralong sonication) is a high yield method, which may induce defects on the material [176]. Additionally, electrochemical exfoliation has recently been reported [173,177,178]. Therefore, even though phosphorene’s properties are believed to be superior than graphene, showing the potential of this 2D material [176] for SIBs It is expected that phosphorene will represent a material of interest for the future of high-performance SIBs once it can deliver over 2000 mAh g^−1^, thereby providing high energy densities for SIBs.

#### 2.4.3. Transition Metal Dichalcogenides 

Similar to phosphorene and graphene (2D materials), transition metal dichalcogenides—also known as TMDs or TMDCs—may provide a two-dimensional layered structure with desirable applications for several research fields, such as energy storage, catalysis, lubrication, nanoelectronics, and optoelectronics [179,180,181]. Their chemistry is versatile-40 different types of layered TMDs have been identified, making TMDs a material that may be able to surpass graphene when it comes to 2D materials [182,183,184].

One of the most commonly studied TMD materials is molybdenum disulfide (MoS_2_), whose structure was reported for the first time in 1923, even though it is believed to have existed on earth for over 2.9 billion years. Furthermore, one of the first works that reported MoS_2_’s layered structure was published in 1986 [185,186]. Layered MoS_2_ was first reported an anode for SIBs in 2013 [187]. The reactions for sodiation, which are analogous to Li-based materials, are expressed by Equation (3). Moreover, the conversion reaction is expressed by Equation (4) [188,189]. MoS_2_ composite materials present high efficiency and capacity for acid functionalized MoS_2_ in graphene oxide [190]. This work, published by David et al., presented a charge capacity of ≈230 mA h g^−1^ at 25 mA g^−1^ with efficiency of ≈99%. Other examples of MoS_2_ with carbon allotropes, for SIB, are MoS_2_ nanosheets aligned with carbon paper. This work concluded that the carbon assures the electronic conductivity of material [191]; and MoS_2_/graphene, synthesized with phosphomolybdic acid (PMA), L-cysteine (C_3_H_7_NO_2_S), and graphene oxide, the latter being reduced by the H_2_S from the L-cysteine molecule [192]. The results from electrode sample MG-3 showed that after 300 cycles, the retained capacities of Na^+^ storage were equal to 254 mAh g^−1^ and 227 mAh g^−1^ at 80 mA g^−1^ and 320 mA g^−1^, respectively [192,193]. Importantly, there are works [188,194] that state that the sodiation peak that occurs at 1.4 V in cyclic voltammograms (CV) happens due to the intercalation of sodium ions within MoS_2_ layers, following the reaction presented by (3):

MoS_2_ + xNa^+^ + xe^−^ ← − → Na*_x_*MoS_2_. (3)

On the other hand, the peak occurring at 0.7 V is characteristic of the reaction presented by (4):

Na*_x_*MoS_2_ + (4 − x)Na^+^ + (4 − x)e^−^ ← − → Mo + 2 Na_2_S. (4)

Likewise, one of the reported TMD materials for high performance SIBs is MoTe_2_, which can be obtained through the spray pyrolysis technique from C–MoO*_x_* [193]. This work reports the performance of C@MoTe_2_ and C/MoTe_2_ and enhanced rate capabilities are obtained when carbon is employed, compared to bare MoTe_2_, mainly after ≈ 100 cycles [193].

**Figure 8:**
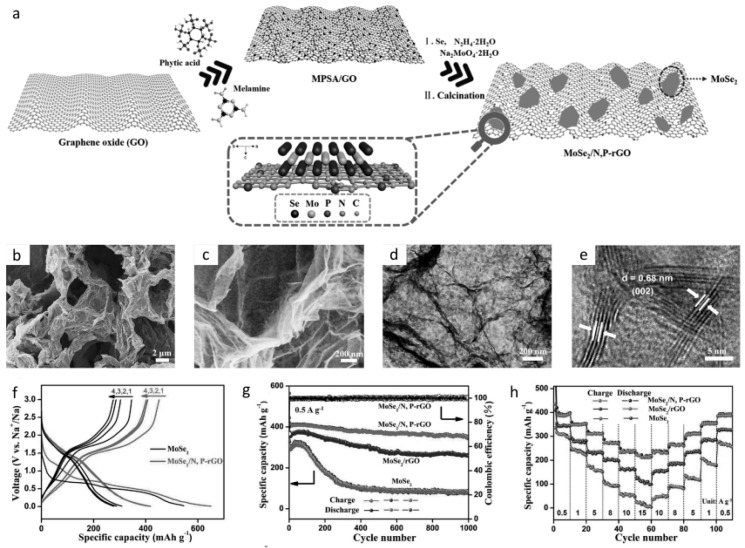
Structural characterization and electrochemical performance analysis of MoSe_2_-covered N,P-Doped carbon nanosheets as anode in SIBs: (**a**) schematics of the synthesis of MoSe_2_/N,P-rGO, (**b**,**c**) scanning electron microscopy (SEM) images of the nanosheets showing the voids achieved in the material, which provide a network capable of withstanding the volume changes and space for electrolyte access, (**d**) transmission electron microscopy (TEM) image of the material showing the MoSe_2_ uniformly deposited on carbon nanosheets, (**e**) high resolution transmission electron microscopy (HRTEM) image of the material with interlayer spacing of approximately 0.68 nm, (**f**) charge/discharge curves of MoSe_2_ pristine and MoSe_2_/N,P-rGO at 500 mA g^−1^, (**g**) cycling performance for current density at 500 mA g^−1^, and (**h**) comparison of the work published by Niu et al. and other works. Reprinted with permission from [196]. Copyright 2017 John Wiley and Sons.

In short, TMDs materials offer a wide variety of composition and physical and chemical properties. When it comes to SIBs, the typical higher interlayer spacing of MoS_2_, for example (approximately 6.15 A), is greater than that of the widely known graphite (approximately 3.3 A), which is an advantage over other material families with respect to expansion upon sodium insertion [195]. Furthermore, these unique chemical and physical properties may provide desirable properties for rate capability, such as the result presented in Figure 8, which includes the findings of Niu et al.’s work [196]. Here, Figure 8a demonstrates the schematic for the synthesis of the TMD composite. Figure 8b–e are the SEM and TEM images of the TMD nanocomposite demonstrating the characteristic morphology under high resolution. Figure 8f–h represent the electrochemical performance of the cell i.e. GCD curves, capacity retention and rate capability. Another point to consider in terms of future perspective and challenges is that new techniques for improved synthesizing nanostructured TMDs are desirable. Moreover, there is a need to develop low-cost and scalable exfoliation techniques that would allow synthesis of TMD nanosheets with ontrolled properties.

#### 2.4.4. MXenes

Transition metal carbides, carbonitrides, and nitrides (MXenes) are 2D materials with a general formula of M_n+1_X_n_T_x_, where M is a transition metal, X can be carbon and/or nitrogen, and T stands for hydroxyl, oxygen, or fluorine. Thus, carbides provide a surface termination [25,197]. This family of materials was discovered in 2011 from the exfoliation of MAX phase materials in hydrofluoric acid (HF) [198]. MXenes are candidates for several applications due to their variety and properties [199,200]. Moreover, due to the fact that MXenes have inter-layer distances larger than graphite, they may enable the intercalation and deintercalation of non-Li-ion batteries, such as SIBs. [200] Examples of MXenes are Ti_3_C_2_, Ti_3_C_2_T*_x_*, Ti_2_C, Ta_4_C_3_, TiNbC, (Ti_0.5_Nb_0.5_)_2_C, (V_0.5_Cr_0.5_)_3_C_2_, and Ti_3_CN [198,201,202].

One of the most studied species for SIB MXene anodes is Ti_3_C_2_T*_x_*. Xie et al. showed that for the first cycle discharge, this material presented a capacity of 370 mAh g^−1^ at 100 mA g^−1^. In contrast, after 120 cycles, the reversible capacity of Na^+^ was 80 mAh g^−1^ [203,204]. In [205], Zhao et al. studied the electrochemical performance of MXene SIBs in 3D macroporous frameworks. By mixing MXene with poly(methyl methacrylate) (PMMA) 3D and making free-standing films of MXene/PMMA, the authors obtained pure MXene by heating the samples up to 450 °C, thereby removing the PMMA. Three kinds of MXenes were synthesized: Ti_3_C_2_T*_x_*, V_2_CT*_x_*, and Mo_2_CT*_x_*. At 0.25 °C, the values of the reversible specific capacity were ≈330 mAh g^−1^, ≈340 mAh g^−1^, and ≈370 mAh g^−1^, respectively. Moreover, the coulombic efficiency after 1000 cycles was close to 100%, and the reversible capacities at 2.5 C were, for Ti_3_C_2_T*_x_*, V_2_CT*_x_*, and Mo_2_CT*_x_*, respectively, 295 mAh g^−1^, 310 mAh g^−1^, and 290 mAh g^−1^ [205].

**Figure 9:**
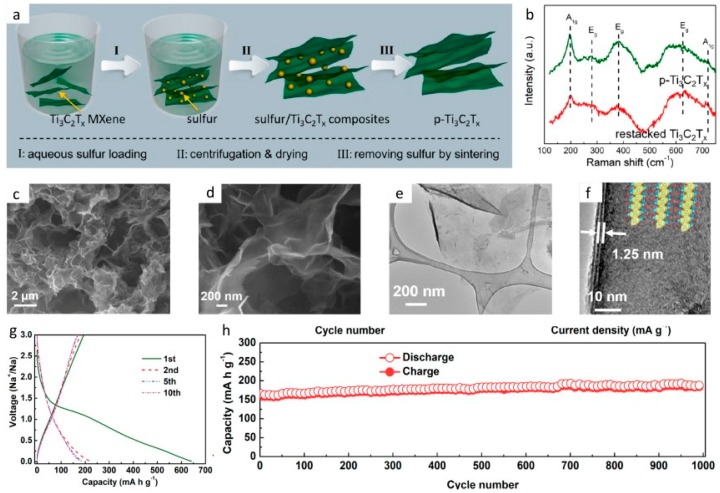
Structural characterization and electrochemical performance analysis of p-Ti_3_C_2_T*_x_* as an anode in SIBs: (**a**) schematic preparation steps of p-Ti_3_C_2_T*_x_*, (**b**) Raman spectra of p-Ti_3_C_2_T*_x_* (green curve) and the pristine restacked Ti_3_C_2_T*_x_* (red curve), (**c**,**d**) scanning electron microscopy (SEM) images of p-Ti_3_C_2_T*_x_* as-prepared, (**e**,**f**) transmission electron microscopy (TEM) of p-Ti_3_C_2_T*_x_* as-prepared, (**g**) charge–discharge curves of p-Ti_3_C_2_T*_x_* 100 mAg^−1^, and (**h**) cycling performance at current density of 1 A g^−1^ showing the cycle stability. Reprinted with permission from [206]. Copyright 2018, American Chemical Society.

Recently, Xie et al. published about a porous Ti_3_C_2_T*_x_* electrode anode for SIBs [206], whose synthesis was performed as presented in Figure 9a. In this work, sulfur addition (being dissolved in ethlyenediamine (EDA)) and its removal after the precipitation of sulfur due to the addition of hydrochloric acid (HCl), provided a porous surface made out of p-Ti_3_C_2_T*_x_* nanosheets on the anode, which enhanced its capacity as well as its rate capability. The p-Ti_3_C_2_T*_x_* presented pore sizes from 1–20 nm and a surface area of 84.2 m^2^ g^−1^. Regarding the electrochemical results, this work reports values for high current densities: for 1000 mA g^−1^, the specific capacitance achieved was 166 mAh g^−1^; while at 10 A g^−1^ it was 124 mAh g^−1^, outperforming works previously published [47,207,208]. Figure 9 includes the important findings of Xie et al.’s work in detail [206]. Figure 9b shows the raman spectra of the MXene before and after restacking, whereas Figure 9c–f are the SEM and TEM images of the layered structure. Figure 9g,h represent the electrochemical performance of the MXene as an anode in SIB.

Thus, MXenes are a promising family of anode materials for SIBs because of their variety, relative stability in dry air, hydrophilicity, and high conductivity [207,209,210,211]. In contrast, the restacking of MXene layers because of van der Waals forces, along with hydrogen bonds, hinders the accessibility of ions to active sites on the materials, thereby decreasing its electrochemical performance [207]. Moreover, mechanical methods for obtaining MXenes are generally not possible due to the strong bonds between layers, which are different from those of TMDs and graphite [197]. Finally, given the fact that MXenes are considered a new family of materials, their properties are yet not fully understood by the scientific community, which means that there are challenges and questions that need to be answered for their wide-scale applicability, and developing methods and strategies for preventing MXene nanosheets from restacking, thereby providing a path for ionic transport.

Table 4 lists the performance of several notable 2D materials which have been used as anodes for Na-ion battery applications.

## 3. Cathodes

Cathodes are a very important aspect of SIBs and should be able to reversibly intercalate the Na^+^ ions, preferably at voltages greater than 2 V [219]. Ideally a cathode should exhibit low volume expansion upon intercalating Na+ ions and structural stability [219]. It is also essential that they can be synthesized from low-cost and earth-abundant precursors and also be able to provide high specific capacities and energy densities [1,22]. Some of the prominent high-performance cathodes currently being studied for SIBs are discussed below in greater detail.

### 3.1. Layered Transition Metal Oxides (TMOs)

Layered TMOs have received significant attention and research as cathodes for LIBs and, therefore, have been applied to SIBs, as well. The most common layered TMO is usually of the form NaMO_2_ (M = Mn, Fe, Co, Ni), which are notable because of their elevated redox potentials and energy densities [19,22]. Consequently, one of the first type of layered metal oxides to be studied for SIBs were of the type AMO_2_, where A = Li, Na and M is a transition metal, e.g., Mn, Fe, Co, etc. [22]. These materials have the advantage of being relatively stable, easy to fabricate, and having a large body of research knowledge already available due to their applications in LIBs for a number of years [220,221]. A study by Delmas et al., published in 1981, compared the performance of different structural variants of Na_x_CoO_2_, the P3 and the O2 [222]. Another important study was by Shibata, who determined the diffusion coefficient of Na^+^ ions into the Na_x_CoO_2_ lattice at room temperatures [223]. Since then, Yabuuchi et al., have synthesized NaFeO_2_ as a cathode for SIB systems [224]. An important study in this area was published by Komaba et al., who studied the performance of Na_2/3_[Fe_1/2_Mn_1/2_]O_2_ as a cathode for SIBs [225,226].

The search for high energy cathode materials has led to the further development of more improved and innovative cathode systems, such as O and the P type layered oxides, where the Na^+^ ion exists in prismatic and octahedral configurations, respectively [19]. Also, depending upon the type of stacking, they are further classified as O3 (ABCABC), P2 (ABBA), and P3 (ABBCCA) types [19,227].

**Figure 10:**
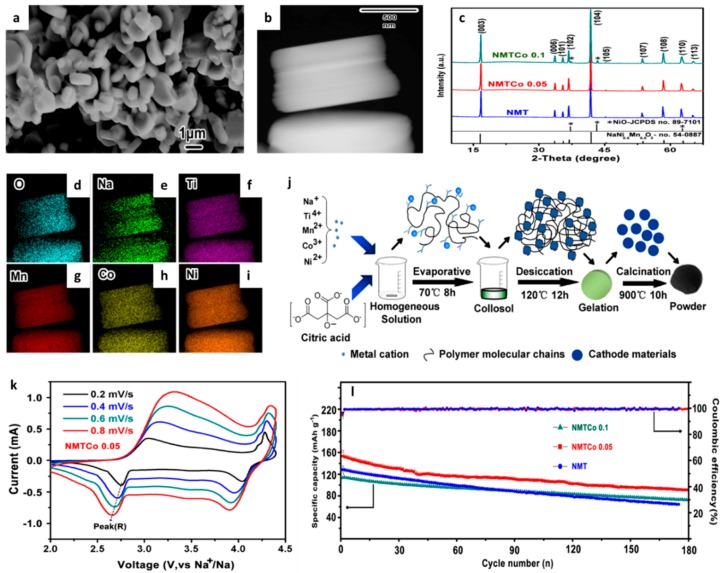
Structural characterization and electrochemical performance analysis of NaNi_0.45−x_Mn_0.25_Ti_0.3_Co_x_O_2_ (NMTCo_x_) cathode in SIBs. (**a**) SEM images of the NMTCo_0.05_ sample demonstrating particle sizes of approximately 1–2 μm with a bead like morphology. (**b**) HRTEM image showing the morphology of the microparticle in greater detail. (**c**) XRD pattern of NMT samples with varying Co concentration (0, 0.05, 0.1) demonstrating the characteristic peaks, strong crystallinity and structural evolution. (**d**–**i**) Elemental maps of the microparticles with the constituent elements labeled. (**j**) Schematic representation of the steps in the synthesis process for the NMT samples. (**k**) Cyclic voltammetry curves of the NMTCo0.05 sample at progressively increasing scan rates (0.2–0.8 mV s^−1^) with the characteristic redox peaks clearly visible. (**l**) Capacity retention and coulombic efficiency plots of the NMT cathodes over 180 cycles. Copyright 2019, American Chemical Society.

A layered quaternary O3 type material, Na(Mn_0.25_Fe_0.25_Co_0.25_Ni_0.25_)O_2_ has been demonstrated to work as a cathode in SIBs by Li et al. [228]. An initial capacity of 180 mAh g^−1^ and an effective specific energy density of 578 Wh kg^−1^ were obtained, which have been attributed to the excellent reversibility of Na+ ions in the O3 layered structure. Zhou et al. have demonstrated that Co substitution enhances the performance of the O3 type material, NaNi_0.45−x_Mn_0.25_Ti_0.3_Co_x_O_2_ (NMTCo_x_), with the optimum x being 0.05 [229]. This cathode provides a specific capacity of 180 mAh g^−1^ at a current density of 10 mA g^−1^. Co enhances the performance by improving electronic conductivity and Na^+^ kinetics [229]. The results of Zhou et al.’s work are provided in Figure 10 below. Here, Figure 10a is a SEM image showing the bead like morphology of the particles with a size range of approximately 1–2 μm, and Figure 10b is an HRTEM image of the same. Figure 10c shows the XRD stack of the various NMTC samples, Figure 10d–i is the elemental maps of the sample, showing a homogenous distribution. Figure 10j is the schematic representation of the synthesis process, and Figure 10k,l is the CV and cycling performance of the material as a positive electrode in SIBs.

A P2 type cathode for SIB applications has been studied by Yabuuchi et al. [226], who synthesized Na_x_[Fe_0.5_Mn_0.5_]O_2_ using a simple solid state reaction from its precursors (Na_2_O_2_, Fe_2_O_3_, and Mn_2_O_3_). A maximum specific capacity of 190 mAh g^−1^ at 2.75 V vs. Na/Na^+^, and an energy density as high as 520 mWh g^−1^, comparable to LiFePO_4_, was obtained. The authors have attributed the beneficial results to the accessibility of the Fe^3+^/Fe^4+^ redox system by intercalating Na^+^ ions. Further, this cathode provides the added advantage of utilizing elements (Fe, Mn) that are abundant in the earth and are, therefore, cost efficient [226]. A high performance P2 type layered Na_0.5_[Ni_0.23_Fe_0.13_Mn_0.63_]O_2_ has been studied by Hasa et al. [230]. Here, too, the electrode was synthesized by a solid state reaction from its precursors and was able to deliver a 200 mAh g^−1^ at a current density of 15 mA g^−1^ with a coulombic efficiency of almost 100%. The stability of the layered structure was deemed responsible for the superior performance [230].

Honeycomb layered Na_3_Ni_2−x_Mg_x_SbO_6_ compounds have been analyzed by You and coworkers. The electrodes were synthesized from their precursors by simple solid state reactions and a maximum capacity of 115 mAh g^−1^ was obtained for the compound with x = 1 [231]. All the cathodes show almost 100% coulombic efficiencies and capacity retentions greater than 70%, even after 200 cycles [231]. A complex phase transition from P3 to O1 during the Na^+^ ion intercalation process and the rearranging of the structure to improve kinetics and stability help to account to for the enhanced performance [231].

Layered TMOs have several advantages. They were one of the first cathodes, e.g., one of the first layered NaMnO_2_ products to be evaluated for application in SIBs, and, therefore, there is a significant body of knowledge available for varying synthesis procedures, especially those related to manipulating the interlayer spacings [19]. An important advantage of these oxides is that they use relatively low cost metals, such as Mn, Fe, Ni, as their constituents [232,233]. These oxides also are able to withstand large strains and demonstrate considerable structural integrity [234].

Despite the above-mentioned performances and advantages, these compounds demonstrate some drawbacks as well. For example, their sensitivity to air and low electronic conductivity is an important issue [19]. Stability is sometimes another issue, with some of the electrodes demonstrating steady capacity decays, especially at high current rates. This is another aspect that needs improvement [219]. Many of these oxides, especially the Mn incorporated ones, tend to undergo a structural distortion—the Jahn–Teller effect that hinders a long cycle life [235].

It is expected that layered TMOs will continue to play a significant role as cathode materials for SIBs. Their easily procurable raw materials, multiple and varying synthesis methods, and different obtainable crystal structures will continue to play an important role in their favor [236]. Several techniques have been developed to overcome the Jahn–Teller distortion, substitution by Mg to suppress the polarization is one of the techniques that have been applied with some success [237]. Also being studied are vanadium based oxides, as the open framework provided by these cathodes allows ions to intercalate easily without much friction or hindrance within the structure, thereby helping to achieve greater stability and reversibility [238].

### 3.2. Polyanionic Compounds

Polyanionic materials provide a novel and diverse group of materials that have found widespread applications as high performance cathodes for SIBs [1]. Various different poly-anionic species, e.g., (PO_4_)^3−^, (SO_4_)^2−^, (P_2_O_7_)^4−^, provide open framework structures that relatively enhance intercalation [1,227].

Sodium Superionic Conductors (NASICON) are a special class of polyanionic compounds that have 3D structures and large tunnels for the fast conduction of Na^+^ ions [19,227]. A classical NASICON compound, Na_3_V_2_(PO_4_)_3_, has a VO_6_ octahedra and PO_4_ tetrahedra sharing a corner, thereby creating a 3D V_2_P_3_O_12_ backbone, as well as multiple Na^+^ ion interstitial sites [239]. Consequently, NASICONs have demonstrated promise as cathodes for SIBs.

#### 3.2.1. Phosphates

NaFePO_4_ have been synthesized and studied as an analogue, keeping the more popular and established LiFePO_4_ electrode in mind, which is generally used for LIBs. The initial synthesis of electrochemically active LiFePO_4_ was performed by low temperature Li/Na ion exchange from LiFePO_4_ [240]. However, the NaFePO_4_ produced by this technique possess weak kinetics. An early work by Lu et al. proposed that for Na_x_FePO_4_, the solid solution phase was preferable for x > 2/3, whereas phase separation into FePO_4_ and Na_2/3_FePO_4_ occurs at x < 2/3 [241]. Further important work in this area was done by Boucher et al., who first revealed the structure of Na_2/3_FePO_4_ using transmission electron microscopy (TEM) and synchrotron x-ray diffraction techniques [242]. A more recent, but pioneering, work in this area was done by Kim et al., who showed that maricite, an electrochemically inactive phase of NaFePO_4_, is electrochemically active when it is made of nanoparticles. A reversible capacity of 142 mAh g^−1^ was obtained, with 95% capacity retention over 200 cycles of operation [243]. More discussion of some of the important works in this area is provided in the paragraphs below.

Sun et al. have studied NaFePO_4_ as a reversible cathode material in SIBs [244]. An olivine electrode was synthesized using a galvanostatic reduction process and was able to provide a capacity of 125 mAh g^−1^ at a working voltage of 2.7 V with a current density of 7.5 mA g^−1^. Structural stability was maintained over 50 cycles of operation, and the authors have attributed this to the rather strong P–O polyanion bonds, which help to preserve the olivine structure [244]. Li et al. have studied hollow amorphous NaFePO_4_ nanospheres as positive electrodes in SIBs [245]. The spheres were prepared using a “hard template method” using iron (II) stearate, NaH_2_PO_4_, and sodium oleate as precursors. A maximum of 152 mAh g^−1^ was obtained at a 1 C current density with more than 95% capacity retention over 300 cycles of operation [245]. The amorphous nature prevents lattice stresses and provides a non-disruptive conductive pathway for the sodium ions, which, as per the authors, enables superior electrochemical performance [245].

**Figure 11:**
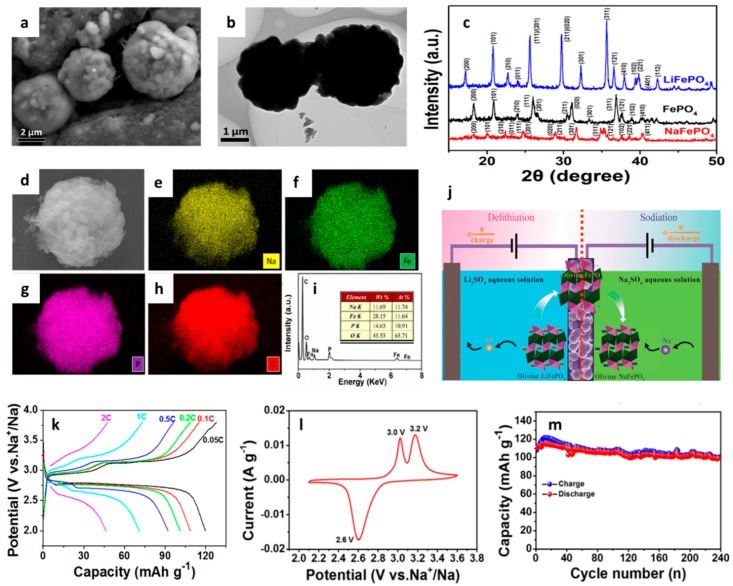
The structural characterization and electrochemical performance analysis of NaFePO_4_/C composite as a cathode in SIBs. (**a**) SEM images of the NaFePO_4_/C composites demonstrating a rather smooth spherical morphology. (**b**) TEM image showing the morphology of the microsphere composite in greater detail. (**c**) XRD pattern of LiFePO_4_ powder precursor, the delithiated FePO_4_ sample and the NaFePO_4_ sample demonstrating the characteristic peaks. (**d**–**h**) Elemental maps of the microsphere composite exhibiting the constituent elements and a rather uniform distribution. (**j**) Schematic representation of the electrochemical displacement reaction that results in the formation of NaFePO_4_ from LiFePO_4_. (**k**) Galvanostatic charging-discharging curves of the half-cell with NaFePO_4_/C cathode at various rates. (**l**) CV of the NaFePO_4_/C cathode system vs. Na/Na^+^ showing the characteristic redox peaks. (**m**) Capacity retention of the PB composite cathode for both the charge and discharge cycles. Copyright 2015, American Chemical Society.

NaFePO_4_ has also been used as a composite and/or a hybrid to obtain enhanced performance. A polythiophene (PTh) encapsulated NaFePO_4_ as a cathode in SIBs has been analyzed by Ali and coworkers [246]. The NaFePO_4_ olivine was synthesized by delithiating LiFePO_4_, followed by in-situ polymerization and the addition of NaI [246]. A specific capacity of 142 mAh g^−1^ was obtained at a current density of 10 mA g^−1^ within a voltage range of 2.2–4.0 V and with a capacity retention of 94% over 100 cycles of operation. The addition of the PTh layer enhanced performance by improving conductivity and enabling better stress relieving of the composite [246]. Along similar lines, Fang et al. synthesized a NaFePO_4_/C microsphere hybrid by forming LiFePO_4_ using lithium acetate, Fe(NO_3_)_3_·9H_2_O, NH_4_H_2_PO_4_, and citric acid as precursors, followed by electrochemical delithiation and subsequent sodiation in a 1 mol l^−1^ Na_2_SO_4_ solution [247]. The olivine cathode was able to provide a specific discharge capacity of 111 mAh g^−1^ with a capacity retention of 90% of ~95% after 240 cycles of operation, at a current density of 0.2C [247]. The authors have been able to identify a two-stage phase transition of NaFePO_4_ during the Na^+^ ion intercalation-deintercalation process [247]. Figure 11 demonstrates the important findings of Fang et al.’s work in greater detail, in which Figure 11a,b features SEM and TEM images, which demonstrate the morphology in greater detail. Figure 10c represents the XRD patterns of the various phosphate phases, and Figure 11d–i shows the elemental maps and elemental composition of the different component elements. Figure 11j features a schematic representation of the electrochemically mediated displacement reaction used to synthesize the NaFePO_4_ sample, and Figure 11k–m shows the representations of the electrochemical performances of the sample positive electrode.

#### 3.2.2. NASICON Compounds and Fluorophosphates

Hong and Goodenough first proposed the tunnel structure for Na^+^ diffusion in 3D Na_1+x_Zr_2_P_3−x_Si_x_O_12_ [248]. Van Alpen proposed a more accurate formula for NASICON for the first time in 1981, and the first non-stoichiometric single crystal rhombohedral NASICON compound, Na_3.1_Zr_1.78_Si_1.24_P_1.76_O_12_, was developed in 1983 [249,250]. Most of the early work focused on synthesizing NASICON electrodes by high temperature solid state reactions [251]. An important development was the synthesis of NASICON from the solutions of their precursors using sol-gel chemistry, and Engell developed several different NASICON compounds using the respective metal alkoxides in about 1983 [252]. However, the first experimental report for the application of NAISCON in energy storage devices was by Delmas et al., who studied NaTi_2_(PO_4_)_3_ as early as 1987 [253]. Since then, NASICON type compounds have been extensively studied and some of the important studies highlighting the applicability of NASICON as positive electrodes in SIBs are provided below.

Kang and coworkers studied a Na_3_V_2_(PO_4_)_3_/C composite as a cathode in SIBs [254]. The cathode was synthesized using a polyol mediated pyro-synthetic method from its precursors. The authors were able to obtain about 117 mAh g^−1^ at 0.01 C and 65 mAh g^−1^ (56% of theoretical capacity) was obtained at a high current density of 2.67 C. The porosity of the nanoparticles and the introduction of C helped to improve the electrochemical performance [254]. Electrospun Na_3_V_2_(PO_4_)_3_/C nanofibers were studied as SIB cathodes by Liu et al. [255]. The precursors were NaH_2_PO_4_, NH_4_VO_3_, citric acid, and polyethylene oxide (PEO), which were electrospun, followed by carbonization (in Ar at 500 °C) and vacuum annealing. 94 mAh g^−1^ was obtained at a current density of 2 C and showed very good stability over 66 cycles of operation [255]. The enhanced performance of NASICON has been attributed to its stability and the uniform contact of the fibers of the composite [255].

A 3D graphene decorated NaTi_2_(PO_4_)_3_ (NTO@rGO) was studied as a cathode in SIBs by Fang et al. [256]. Reversible capacities as high as 138 mAh g^−1^ at 0.1 C and a capacity retention of 77% over 1000 cycles were obtained, attributable to the 3D structure and improvement of conductivity by graphene addition [256]. Gao et al. examined NASICON structured Na_3_MnZr(PO_4_)_3_ for applications as positive electrodes in SIBs [257]. The material was prepared using a sol-gel technique and provided a specific discharge capacity of 105 mAh g^−1^ at a current rate of 0.1 C (which was almost equal to the theoretical capacity of 107 mAh g^−1^), which the authors have attributed to the reversible intercalation/deintercalation of Na^+^ ions into the NASICON lattice [257]. However, at higher C rates, performance decreased due to Jahn–Teller distortion and oxidation of the electrolyte [257].

**Figure 12:**
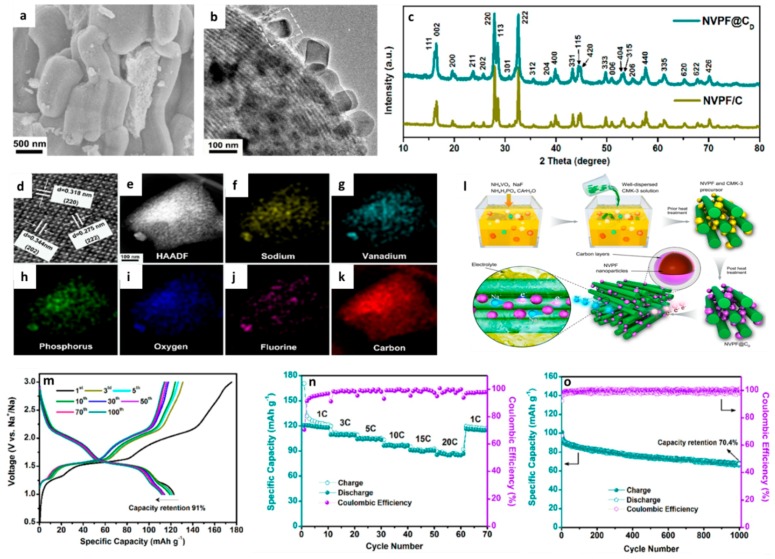
Structural characterization and electrochemical performance analysis of NaFePO_4_/C composite as a cathode in SIBs. (**a**) SEM images of the Na_3_V_2_(PO_4_)_2_F_3_@C (NVPF/C) composites exhibiting bead like morphology. (**b**) TEM image showing the morphology demonstrating the “worm-like” mesoporous structure. (**c**) XRD pattern of NVPF sample with the core-double shell structure (NVPF@C_d_) in comparison with the NVPF sample with just simple carbon coating (NVPF@C), demonstrating the characteristic peaks. (**d**) FFT image of the NVPF@Cd sample clearly demonstrating the carbon coating in the nanocomposite. (**e**–**h**) Elemental maps of the NVPF@C_d_ composite depicting the constituent elements uniformly distributed. (**j**) Schematic representation of the synthesis of the NVPF@Cd nanocomposite from its precursors. (**k**) Galvanostatic charging-discharging curves of the half-cell with NVPF/C_d_ cathode for various cycles. (**l**) Rate performance demonstration of the nanocomposite electrode showing its performance at varying C rates. (**m**) Capacity retention of the NVPF@C_d_ composite and its corresponding coulombic efficiency. Copyright 2016, American Chemical Society.

Liu et al. studied a core shell structured fluorophosphate nanocomposite, Na_3_V_2_(PO_4_)_2_F_3_@C, (NVPF@C) as a cathode in SIBs [258]. A reversible specific capacity of 125 mAh g^−1^ was obtained at a current density of 0.5 C, 84 mAh g^−1^ at high current densities, such as 50 C, and the material showed a 65% capacity retention, even after 5000 cycles. This high performance and stability could be related to the confinement of the NVPF particles in the mesoporous structure [258]. The important results of Liu et al.’s work are summarized in Figure 12 below. Here, Figure 12a,b is the SEM and TEM images, respectively, of the composite bead, which shows characteristic morphology. Figure 12c shows the XRD patterns of the core shell sample compared to that of the just carbon coated sample. Figure 12d represents the HRTEM FFT image of the core shell sample, and Figure 12e–k shows the elemental maps. Figure 12i schematically represents the synthesis procedure and Figure 12m–o demonstrates the electrochemical analysis of the material as a positive electrode in SIBs.

However, the presence of these large polyanionic groups reduces net energy density, and NASICON compounds need carbon or graphene support to improve conductivity and kinetics. Thus, greater work needs to be done to enhance their effectiveness.

Polyanionic materials, especially NASICONs, have attracted significant attention due to their large networks and variable tunnel structures. They are able to easily allow insertion and deinsertion of Na+ ions and are relatively structurally stable, thereby allowing for a long cycle life [259]. Notably, NASICONs exist in an assortment of structures, from orthorhombic Na_3_Ti_2_(PO4)_3_, to rhombohedral, to the monoclinic Na_3_V_2_(PO4)_3_ [260].

However, low electronic conductivity is one of the primary hindrances to realizing their full potential. Surface functionalization with carbon and making nanoparticles seemed to improve the performance in this case [19,261]. Nitrogen doping has been also demonstrated to improve overall electrochemical performance, but it has been observed that N doped materials are particularly sensitive to moisture, which reduces their effectiveness [262]. The use of vanadium ions in the NASICON structure has also raised toxicity and environmental concerns [263].

The path forward for NASICONs lies in integrating them with high conductivity materials, such as composites with graphene or carbon nanotubes, thereby making nanostructures to improve conductivity and reduce structural defects during synthesis [264,265]. With the advent of sol-gel based techniques for NASICON synthesis, greater control can be had over the structural parameters, and this promises an important path forward [266].

### 3.3. Prussian Blue

Prussian blue (PB), chemically, is a compound with a mixed valency of iron, generally in the +2 and +3 oxidation states. Fe_4_[Fe(CN)_6_]_3_·nH_2_O is the most general formula of an unmodified PB compound [267]. The crystal’s structure is usually cubic, with iron atoms alternating at the face centered position associated with C–N pairs and the Fe^3+^ and Fe^2+^ being surrounded by nitrogen and carbon environments, respectively; this compound can be chemically referred to as a hexacyanoferrate (HCF). There are zeolitic water molecules that are generally contained in some of the octahedral vacant interstitial spaces. Essentially, due to its mixed-valence and open framework structure, PB can be modified by other atoms like Mn, Zn, Ni, Co substitutionally for Fe, and these compounds are then designated as prussian blue analogues (PBA). These PBAs provide channels for alkali ion diffusion, and the choice of the transition metal substituting for Fe can enhance the intercalating ionic mobility further by reducing the diffusion barrier. These interesting properties have resulted in significant interest in PBs and PBAs as cathode materials for SIBs.

PB and PBAs were known in historical times, especially for their applications as pigments [268]. For their application in energy storage devices, they were first studied as Li^+^ ion carries in aprotic media [269]. However, a capacity loss of about 30%–40% was observed after just 10 cycles, resulting in further research on their structure and properties [267,269]. The first significant result using PBAs as a cathode for SIBs was published by Wessels et al., who showed a relatively stable performance of copper hexacyanoferrate electrodes in aqueous media for as many as 40,000 cycles [270]. Another important milestone in the development of PB electrodes is the fabrication of low and high quality PBAs by You et al., which demonstrated the role of inherent structural defects and how careful synthesis can improve overall stability and cycle life performance [271]. There has been significantly more research on PBAs since then, and the discussion below focuses on some of the important results.

**Figure 13:**
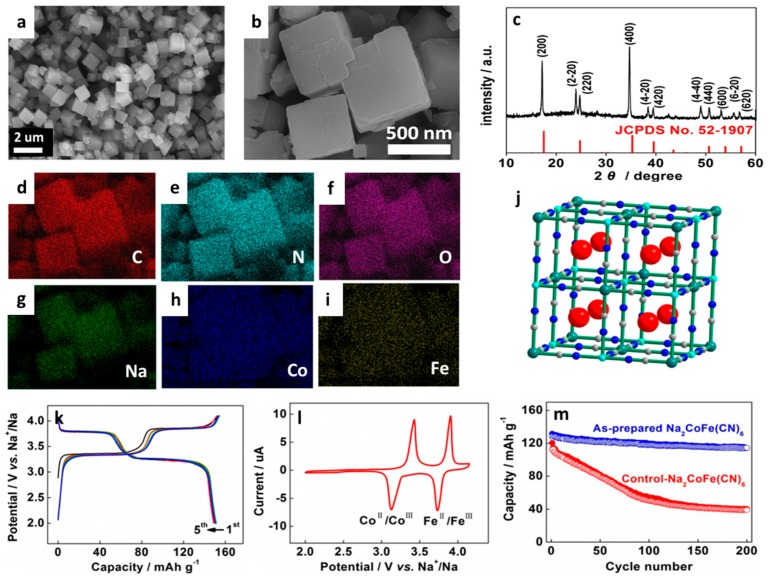
Structural characterization and electrochemical performance analysis of Prussian blue (PB) as a cathode in SIBs. (**a**,**b**) SEM images at low and high magnification of the sodium rich PB crystals depicting an uniform cubical morphology. (**c**) XRD pattern of the synthesized PB sample demonstrating high degree of crystallinity. (**d**–**i**) Elemental mapping of the PB sample demonstrating the different constituent elements with uniform distribution. (**j**) Schematic representation of the PB crystal showing the FCC (face centered cubic) lattice structure, with the Na atoms occupying the interstitial red sports. (**k**) Galvanostatic charging-discharging curves of the half-cell with PB cathode. (**l**) CV of the PB cathode system vs. Na/Na^+^ showing the characteristic redox peaks (**m**) Capacity retention of the PB cathode compared with a control, which is a more defect-filled PB structure. Copyright 2016, American Chemical Society.

Lu et al. studied a PB system as a cathode in SIBs [272]. They prepared PB analogues with the general formula, KMFe(CN)_6_ (M = Mn, Fe, Co, Ni, Zn), at room temperature using a substitutional reaction with a salt solution of the “M” constituent [272]. A discharge capacity of more than 100 mAh g^−1^ was obtained at a C/20 discharge rate with high capacity retention (>95%) after 30 cycles of operation [272]. However, coulombic efficiencies were low (~60%–65%); these low efficiencies have been attributed to interstitial water in the PB analogues [272]. Similarly, You et al. studied “high-quality” (HQ) PB crystals as cathodes in SIBs [271]. Their HQ PB sample had the stoichiometry of Na_0.61_Fe[Fe(CN)_6_]_0.94_ and was prepared by reacting a Na_4_Fe(CN)_6_ precursor with 37% HCl. A specific capacity of 170 mAh g^−1^ and a coulombic efficiency of ~100% were obtained after 150 cycles of operation [271]. Li et al. analyzed a sodium rich prussian blue framework, Na_1+x_FeFe(CN)_6_, as a cathode in an SIB system [273]. The PB framework was prepared in a facile, one-step synthesis process using Na_4_Fe(CN)_6_ and NaCl as the precursor. A maximum of 103 mAh g^−1^ was obtained at a current density of 20 mA g^−1^, with a capacity retention of 97% over 400 cycles [273]. The authors have proposed a low crystalline water content and low vacancies as being responsible for the high stability [273]. A mesoporous PB analogue, KNiFe(CN)_6_, synthesized by a template-free technique, was analyzed by Yue et al. [274]. A reversible specific capacity of 65 mAh g^−1^ at a low current density of 10 mA g^−1^ was obtained with a good rate capability, which, according to the authors, was due to the crystallinity and porosity of the sample [274]. A citrate controlled reaction mechanism was used by Wu and coworkers to synthesize a low-defect PB analogue using CoCl_2_, Na_3_C_6_H_5_O_7_, and Na_4_Fe(CN)_6_ as precursors [275]. A specific capacity of ~150 mAh g^−1^ and 90% capacity retention were obtained over 200 cycles [275]. Results from Wu’s work are demonstrated in Figure 13. Figure 13a,b shows the SEM images of the prussian blue cubes at low and high magnification, whereas Figure 13c represents the XRD pattern of the PB sample. Elemental maps of the sample are demonstrated in Figure 13d–i, while Figure 13j is the schematic representation of the PB cube. Figure 13k–m shows the electrochemical analysis of the cell.

The large vacant spaces and open network based 3D structure makes PBs interesting and attractive cathode materials [271]. The effective choice of a transition metal during its synthesis helps in stabilizing its cubic structure further [276]. Because of the presence of these vacant open spaces, they can easily intercalate large alkali ions, such as Na^+^, K^+^ without much difficulty, thereby satisfying one of the key criteria of SIB cathodes, i.e., their ability to smoothly and reversibly intercalate the bulky Na^+^ ion [277]. A large number of PB analogues have been synthesized, especially within the last decade [278,279].

These advantages notwithstanding, PB suffers from several important shortcomings. Primary among these is the presence of significant amounts of interstitial or zeolitic water. It has been estimated that interstitial water is more than 15 wt.%, and this significantly hinders the long term cycle life by occupying electrochemically active sites [280]. Secondly, it has been found that, for the Fe incorporated PBs (which are also the most common), only the C coordinated Fe sites are electro-active, indicating that half of the vacant lattice spaces are essentially unused [274,281]. However, later research, with the help of X-ray absorption near edge structure (XANES) and extended X-ray absorption fine structure (EXAFS) analysis, have also demonstrated that the Cu or the Co edge is also electrochemically active if the compound is CuHCF or CoHCF [282,283]. Results have also shown that the co-precipitation techniques of synthesizing PBs result in approximately 30% of the lattice vacancies being unavailable due to structural defects and distortion (wrapping). Also, it can be observed that the performance of some of the PBAs are pH dependent (e.g., CuHCF), which limits its performance [284].

A significant amount of research is focusing on PBs to be able to overcome these problems. defect free, mesoporous, and other high-surface area Pb analogues are being synthesized to improve their electrochemical activity [285]. Zeolitic water elimination has been achieved by Goodenough by synthesizing rhombohedral PB analogue, having only 0.08 moles of H_2_O rather than the usual cubic structure [286]. Overall, it is understood that all the various PB frameworks may not be perfectly suitable cathodes for SIBs. PB analogues have provided some interesting outcomes when Zn^2+^ is used as the intercalating ion instead of alkali ions [287]. The use of surface functionalized PB analogues and applications in low cost aqueous systems are some of the areas in which PB cathodes look to find future applications [288].

### 3.4. Organic Materials

The research into organic electrodes for SIBs goes back approximately 30 years. Carbonyls based on the C=O bond have been the predominant electrodes that have found applications in SIBs [289]. However, some of the carbonyls exhibit insulating behavior, which renders them ineffective and has spurred the necessity for further research into other organic compounds as positive electrodes for SIBs [290,291]. Quinone compounds are some of the most popular and earliest materials that have been studied as cathodes for SIBs [292]. Following quinones, several other classes of compounds have been studied, such as polyimide and composites of organic molecules, e.g., aniline/o-nitroaniline co-polymers, as studied by Zhao et al. [293,294]. There has been further research in this area, and some prominent studies are discussed below.

A low cost polymeric cathode material, p-dopable polytriphenylamine (PTPAn), has been studied in SIBs by Deng et al. [295]. When coupled with an n-type poly(anthraquinonyl sulfide) (PAQS) anode, this all-organic SIB delivered a maximum reversible capacity of 132 mAh g^−1^ at a 1 C current rate and a specific energy of 55 Wh kg^−1^ [295]. The electrodes were also able to retain 85% of their specific capacity over 500 cycles and demonstrated a coulombic efficiency of ~99% [295]. Yao et al. analyzed the feasibility of indigo carmine as a positive electrode material in SIBs [296]. An optimum specific capacity of 100 mAh g^−1^ was obtained at a voltage of 1.8 V vs. Na^+^/Na for the material. Approximately 80% of the initial capacity was retained after 40 cycles of operation at a current density of 10 mA g^−1^ [296].

**Figure 14:**
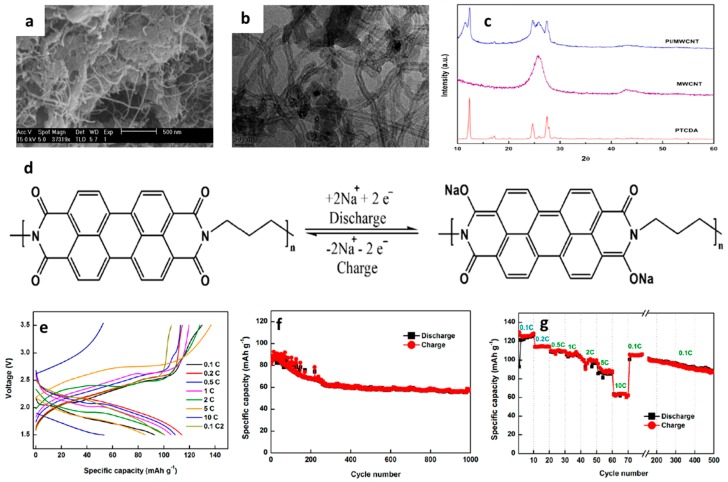
Structural characterization and electrochemical performance analysis of PI/MWCNT composite as a cathode in SIBs. (**a**) SEM images demonstrating a 3D network of fibers as the morphology of the PI/MWCNT fibers. (**b**) TEM image exhibiting the fiber network in greater detail. (**c**) XRD pattern of the PI/MWCNT fiber composite in comparison with its precursors with the characteristic peaks being visible. (**d**) Schematic reaction map depicting the synthesis of the PI/MWCNT nanocomposite. (**e**) Galvanostatic charge–discharge graphs of the PI/MWCNT composite as cathode in the SIB. (**f**) Capacity retention for the composite cathode for both charge and discharge cycles. (**g**) Rate performance of the PI/MWCNT cathode demonstrating almost all capacity retention when the rate is switched back to lower values. Copyright 2018, American Chemical Society.

Polyimides (PIs) derived from aromatic carbonyl precursors as cathode materials in SIBs were analyzed by Wang and coworkers [297]. The PIs obtained from Perylene-3, 4, 9, 10-tetracarboxylic acid-dianhydride (PTCDA) performed the best, with specific power and energy capacities of 20.99 kW kg^−1^ and 285 Wh kg^−1^, along with a capacity retention of 87.5% of the initial capacity over 5000 cycles of operation [297]. Along similar lines, Luo et al. studied commercially obtained PTCDA directly as a cathode material in SIBs [298]. They were able to obtain a reversible capacity of 145 mAh g^−1^ along with the ability to provide 91 mAh g^−1^ at a very high current of 1000 mA g^−1^ and a stable cycling performance [298]. PTCDA was also used as the precursor material to obtain PI, which was then hybridized with MWCNTs for application as cathodes in SIBs, by Manuel et al. [299]. A maximum discharge capacity of 125 mAh g^−1^ was obtained at a 0.1 C rate, along with 100% capacity retention and 100% coulombic efficiency after 70 cycles of operation [299]. The results of this work have been provided in Figure 14. In this, Figure 14a,b shows the SEM image of the 3D network of the PI/MWCNT composite and Figure 14c shows their XRD pattern. The arrangement of the composite is shown schematically in Figure 14d. Figure 14e–g represents the electrochemical performance of the composite.

Organic electrodes have the advantage of being composed of relatively inexpensive precursors [300,301]. Organic electrodes synthesized from quinone and pteridine generally exhibit high capacities [302]. Organometallic polymers demonstrate high rate capacities and kinetics, whereas polyimide electrodes provide high stability (approximately 500 cycles) [303]. However, many organic electrodes suffer from low conductivity and poor coulombic efficiencies. Dissolution in the electrolyte is another area of concern for organic electrodes [219].

Future work in organic electrodes should aim to design the molecule to incorporate a combination of the advantages of its various components, essentially creating a composite. For example, the theoretical capacity of quinone can be fully realized by developing a composite of it with polyimide, thereby taking advantage of the latter’s long term stability [219,304]. The conductive coating and surface functionalization (with polyaniline) of low conducting polymers is another area that is being explored to enhance the performance of these materials [305]. Considering the relatively cheap cost of precursors, organic electrodes have the ability to be an important class of cathode materials for SIBs.

A tabular listing of some other prominent results for the cathodes discussed above is provided in Table 5 below.

## 4. Conclusions

SIBs are considered to be a highly promising alternative to LIBs. However, in order for that goal to be achievable in the near future, electrode materials must be tailored to provide high energy and power densities. Further the electrode materials must also prove durable and provide a significantly long cycle life, especially since SIBs are being considered for grid scale storage, where reliability and longevity are two of the most important parameters.

It is here that these high-performance materials play a very important role. On the anode side, novel 2D materials like TMDs and layered materials like MXenes have the ability to provide large surface areas and enhanced electronic conductivity, as well as access to a greater number of electrochemical sites. Likewise, considering the cathodes, novel materials like NASICON and polyanionic compounds, PB analogues, etc., are currently at the forefront of SIB technology. These materials provide large open framework structures, thereby allowing easy intercalation of Na^+^ ions and less structural degradation during the intercalation/deintercalation process.

However, considerable challenges still exist in all these cases. Layered materials tend to restack after several cycles, which necessitates using support materials within the layers, essentially making the synthesis complicated. Also, the polyanionic compounds tend to suffer from low electronic conductivity, which also makes a graphene/CNT coating necessary.

Regardless of these challenges, greater research in this area will help to elucidate and improve the understanding of the electrochemical mechanisms involved. This will go a long way in adopting SIBs for commercial purposes and greatly enhancing their feasibility.

## Figures and Tables

**Table 1:** High performance of carbon-based anodes in Na-ion battery systems.

Type of Carbon Anode	Electrolyte Chemistry	Voltage Range (V)	Performance *	Reference
Natural Graphite	1 M NaPF_6_ in DEGDME (-)	0.01–3.00	150/2500/100	[53]
Carbon nanosheets derived from peat moss	1 M NaClO_4_ in EC/DEC (1:1)	0.01–3.00	255/210/100	[54]
Soft Carbon	1 M NaPF_6_ in EC/DEC (1:1)	0.01–2.00	114/300/1000	[33]
Nitrogen doped porous carbon	1 M NaClO_4_ in PC (-)	0.01–3.00	243/100/50	[55]
Hollow carbon nanospheres	1 M NaClO_4_ in PC (-)	0.01–3.00	160/100/100	[56]
Hard carbon micro-spherules	1 M NaClO_4_ in EC/DEC (1:1)	0.01–3.00	290/100/30	[57]
Wood fiber derived hard carbon	1 M NaClO_4_ in EC/DEC (1:1)	0.01–2.5	196/200/100	[58]
Hard-soft composite carbon	1 M NaClO_4_ in EC/DEC (1:1)	0.01–2.5	191/100/150	[59]
Hard carbon microtubes (HCTs)	0.8 M NaPF_6_ in EC/DMC (1:1)	0.01–2.5	305/100/30	[60]
Microstructure-controlled amorphous carbon	1 M NaPF_6_ in EC/DMC (1:1)	0.01–3.00	190/200/300	[61]

* Specific capacity (mAh g^−1^)/number of cycles/current density (mA g^−1^). DEGDME = Diethylene glycol dimethyl ether, EC = Ethylene carbonate, DEC = Diethyl carbonate, PC = Propylene carbonate, DMC = Dimethyl carbonate.

**Table 2:** High performance of alloy-based anodes in Na-ion battery systems.

Type of Alloy-Based Anode	Electrolyte Chemistry	Voltage Range (V)	Performance *	Reference
Sn coated viral nanoforests	1 M NaClO_4_ in EC/DEC (1:1)	0.01–1.5	405/150/50	[65]
Sn nanofibers	1 M NaClO_4_ in PC with 2% FEC	0.001–0.65	776.26/100/84.7	[103]
Sb/MWCNT (Multi-walled carbon nanotube)	1 M NaClO_4_ in PC (1:1) with 5/10% FEC	0.01–2.5	~382/120/200	[104]
Sb nanocrystals	1 M NaPF_6_ in EC/DMC (-)	0.02–1.5	~550/100/660	[93]
Sb@C microspheres	1 M NaPF_6_ in EC/DEC (1:1)	0.01–3.00	~584/100/200	[105]
Sb@TiO_2_	1 M NaClO_4_ in PC (1:1) with 5% FEC	0.01–3.00	541/100/100	[106]
Zn_4_Sb_3_	1 M NaClO_4_ in PC (1:1) with 5% FEC	0.01–2.0	290/200/414	[107]
FeSb_2_	1 M NaClO_4_ in PC with 5% FEC	0.01–1.2	540/130/36	[108]
SnSb@carbon nanocable	1 M NaClO_4_ in PC (1:1) with 5% FEC	0.005–1.5	360/100/100	[109]
Si/Ge nanorod	1 M NaPF_6_ in EC/DEC (1:1)	0.001–1.5	20 μAh cm^−2/^200/10 μA cm^−2^	[67]
Bi@C microsphere	1 M NaClO_4_ in EC/PC (1:1)	0.01–2.0	123.5/100/100	[110]
Sn-Bi-Sb	1 M NaClO_4_ in PC (1:1) with 5% FEC	0.01–2.0	621/100/200	[111]
Sn_4_P_3_	1 M NaClO_4_ in EC/DEC (1:1)	0.01–1.5	~700/100/100	[112]
SnP nanocrystals	5 M NaFSI in DME	0.005–1.5	600/200/100	[113]
Cu_2_P/C	1 M NaClO_4_ in EC/DEC (1:1) with 5% FEC	0.01–1.5	430/100/200	[114]

* Specific capacity (mAh g^−1^)/number of cycles/current density (mA g^−1^). FEC = Fluoroethylene carbonate.

**Table 3:** High performance of metal oxides anodes in Na-ion battery systems.

Type of Metal Oxide Anode	Electrolyte Chemistry	Voltage Range	Performance *	Reference
P2-Na_2/3_Co_1/3_Ti_2/3_O_2_	1M of NaClO_4_ in EC/DEC/3 wt.% FEC	4.0–2.0	64.9/400/1C ^a^	[133]
Tunnel-Na_0.44_MnO_2_	1M NaClO_4_ in PC	2–3.8	82/1000/0.42 C ^a^	[134]
Fe_3_O_4_ QD@C-GN	1M NaFP_6_ in EC/DMC	0–3.0	343/1000/2000	[135]
Co_3_O_4_@NC	1M NaClO_4_ in PC/PEC	0–3.0	175/1100/1000	[136]
SnO_2_/3D graphene	1M NaPF_6_ in EC/DEC/10 wt.% FEC	0–2.8	223/350/80	[137]
T-Nb_2_O_5_/CNF	1M NaClO_4_ in PC/EC/5 wt.% PEC	0–2.8	150/5000/1000	[138]
Na_2_Ti_6_O_13_ nanorods	1M NaClO_4_ in PC and FEC	0–2.5	109/2800/1000	[139]
Sb_2_O_3_/Sb@Gr-CSN	1M NaClO_4_ in EC/DMC	0–2.5	487/275/100	[140]
V_2_O_3_/C	1M NaClO_4_ in DMC/EC	0–3.0	181/1000/2000	[141]
3-CCO@C	1M NaClO_4_ in DMC/EC	0–3.0	314/1000/1000	[142]
Inverse opal am-TiO_2_	1M NaPF_6_ in DEG-DME	0–3.0	86.7/500/500	[143]
Nickel-titanium oxide	1M NaPF_6_ in DMC/EC/5 wt.% FEC	0–2.5	491/200/50	[144]
M-Na_4_Ti_5_O_12_/CNT	1M NaClO_4_ in DMC/EC	0.01–3.0	84.4/500/100	[145]
Na_2_Ti_2_O_5∙_H_2_O/MoS_2_-C	1M NaClO_4_ in DMC/EC/5 wt.% FEC	0–3.0	201.1/16,000/8000	[81]

* Specific capacity (mAh g^−1^)/number of cycles/current density (mA g^−1^). ^a^ C rate.

**Table 4:** High performance of 2D materials as anodes in Na-ion battery systems.

Type of 2D Materials Anode	Electrolyte Chemistry	Voltage Range (V)	Performance *	Reference
P/N-doped graphene	1M NaPF_6_ in EC/DEC	-	809/350/1500	[212]
HRGO300	1M NaClO_4_ in EC/DMC/FEC	0–3.0	163/3000/2000	[213]
VSG2	ester-based	0–3.0	402/300/500	[214]
Nanoporous RP on rGO	1M NaClO_4_ in PC/5 wt.% FEC	0.01–2.0	775.3/1500/5120	[215]
SbPO_4_/rGO	1M NaClO_4_ in PC	0–1.5	100/1000/1000	[156]
Ti_2_Nb2O_9_ nanosheets	1M NaPF_6_ in diethylene glycol dimethyl ether	~0.1–3.0	~160/500/800	[216]
MoS_2_/S-doped graphene	1M NaPF_6_ in DEC/EC	0.005–3.0	309/500/1000	[217]
Mesoporous MoS_2_/C	1M NaClO_4_ in DMC/EC/5 wt.% FEC	0.05–3.0	390/2500/1000	[218]
MoSe_2_@N, P-carbon nanosheet	1M NaPF_6_ in DEC/EC/5 wt.% FE	0–3.0	168/1000/500	[196]

* Specific capacity (mAh g^−1^)/number of cycles/current density (mA g^−1^).

**Table 5:** High performance cathodes for SIBs.

Type of Cathode	Electrolyte Chemistry	Voltage Range (V)	Performance *	Reference
	**Layered TMOs**			
Na_0.44_MnO_2_	1M NaClO_4_ in EC:DEC (1:1)	2.0–3.8	80/2000/0.1 C ^b^	[306]
Na_0.67_[Mn_0.65_Co_0.2_Ni_0.15_]O_2_	1M NaPF_6_ in EC:DEC (1:1)	2.0–4.4	129/50/20	[307]
NaMnTi_0.1_Ni_0.1_O_2_	1M NaClO_4_ in PEC	1.5–4.2	186/500/0.1 C ^b^	[308]
Na_x_Al_0.1_Mn_0.9_O_2_	1M NaPF_6_ in PEC	2.0–3.8	160/100/0.1 C ^b^	[309]
Na[Li_0.05_(Ni_0.25_Fe_0.25_Mn_0.5_)_0.95_]O_2_	1M NaClO_4_ in PEC:FEC (1:1)	1.75–4.3	177/200/0.1 C ^b^	[310]
	**Phosphates**			
Mesoporous amorphous FePO_4_	1M NaPF_6_ in EC:DEC (1:1)	1.5–3.8	151/160/20	[311]
Amorphous FePO_4_/CNT	1M NaClO_4_ in EC:DMC (1:1)	2.25–3.75	66/300/50	[312]
NaFePO_4_	NaTFSI/BMP–TFSI (IL)	2.0–3.75	120/100/0.05 C ^b^	[313]
NaFePO_4_	1M NaPF_6_ in EC:DMC (1:1)	1.5–4.0	150/200/0.1 C ^b^	[240]
Na_2_Fe_2_P_2_O_7_	1M NaClO_4_ in PC	2.0–4.0	90/30/0.1 C ^b^	[314]
	**NASICON and Fluorophosphates**			
Na_3_V_2_(PO_4_)_3_	1M NaClO_4_ in PC	2.5–3.8	66.5/50/1 C ^b^	[259]
C coated Na_3_V_2_(PO_4_)_3_	1M NaPF_6_ in EC:DMC (1:1)	2.5–4.0	113/8000/2 C ^b^	[315]
Graphene supported Na_3_V_2_(PO_4_)_3_	1M NaClO_4_ in PC	2.5–3.8	90/100/1 C ^b^	[316]
Fe_2_(MoO_4_)_3_	1M NaClO_4_ in EC:DMC (1:1)	1.5–3.5	94/100/1 C ^b^	[317]
Na_3_V_2_O_2_(PO_4_)_2_F	1M NaClO_4_ in FEC	2.5–4.3	100/1000/20 C ^b^	[318]
	**PB**			
Na_4_Fe(CN)_6_	1M NaClO_4_ in EC/DEC (1:1)	2.0–4.2	100/70/5	[319]
Na_2_NiCo_1−x_Fe(CN)_6_	1M NaPF_6_ in EC/DEC (1:1)	2.0–4.2	124/100/50	[320]
Na_x_CoFe(CN)_6_	1M Na_2_SO_4_ (pH = 7)	0.5–2.0	100/200/600	[285]
K_0.33_FeFe(CN)_6_/rGO	1M NaClO_4_ in EC/DEC (1:1)	2.0–3.8	160/1000/0.5 C ^b^	[321]
Na_0.647_Fe[Fe(CN)_6_]_0.93_.2.6H_2_O/C	1M NaPF_6_ in EC/DEC (1:1)	2.0–4.0	130/200/50	[279]
	**Organic**			
Polyimide (PI)	1M NaPF_6_ in PC	1.5–3.0	180/50/50	[322]
Anthraquinone based PI	1M NaPF_6_ in DME:DOL (1:1)	1.5–3.5	179/150/50	[323]
Poly(benzoquinonyl sulfide) (PBQS)	1M NaTFSI/DOL DME	1.5–4.0	268/1000/50	[324]
Disodium rhodizonate	1M NaClO_4_ in PC	0.0–3.1	498/50/50	[325]
Juglone/rGO	1M NaClO_4_ in EC:DMC (1:1)	0.0–2.5	760/100/100	[290]

* Specific capacity (mAh g^−1^)/number of cycles/current density (mA g^−1^). ^b^ C rate.
